# Effect of Iron Doping in Ordered Nickel Oxide Thin Film Catalyst for the Oxygen Evolution Reaction

**DOI:** 10.1021/acscatal.4c02572

**Published:** 2024-09-11

**Authors:** Ane Etxebarria, Mauricio Lopez Luna, Andrea Martini, Uta Hejral, Martina Rüscher, Chao Zhan, Antonia Herzog, Afshan Jamshaid, David Kordus, Arno Bergmann, Helmut Kuhlenbeck, Beatriz Roldan Cuenya

**Affiliations:** Department of Interface Science, Fritz-Haber Institute of the Max Planck Society, Faradayweg 4-6, Berlin 14195, Germany

**Keywords:** model catalysts, alkaline OER, NiO thin films, Fe doping, *operando* spectroscopy

## Abstract

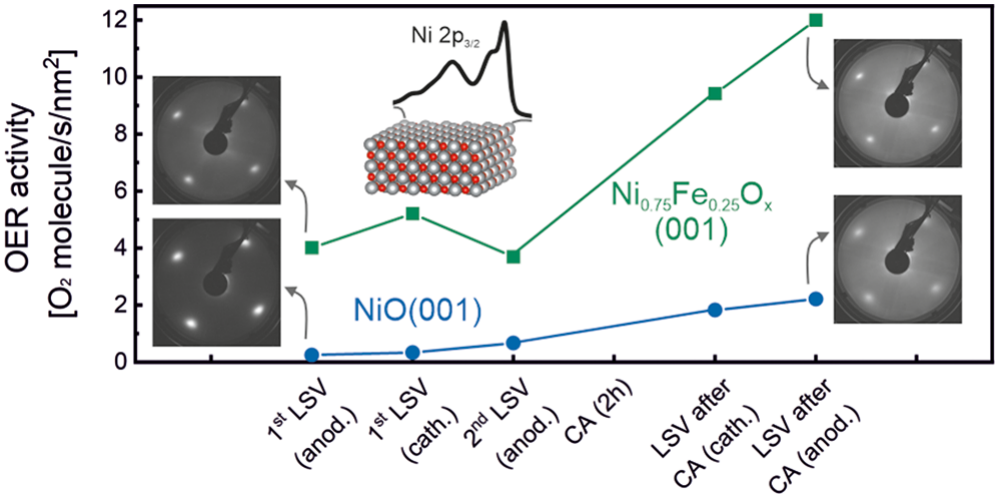

Water splitting has emerged as a promising route for generating hydrogen as an alternative to conventional production methods. Finding affordable and scalable catalysts for the anodic half-reaction, the oxygen evolution reaction (OER), could help with its industrial widespread implementation. Iron-containing Ni-based catalysts have a competitive performance for the use in commercial alkaline electrolyzers. Due to the complexity of studying the catalysts at working conditions, the active phase and the role that iron exerts in conjunction with Ni are still a matter of investigation. Here, we study this topic with NiO(001) and Ni_0.75_Fe_0.25_O_*x*_(001) thin film model electrocatalysts employing surface-sensitive techniques. We show that iron constrains the growth of the oxyhydroxide phase formed on top of the Ni or NiFe oxide, which is considered the active phase for the OER. Besides, *operando* Raman and grazing incidence X-ray absorption spectroscopy experiments reveal that the presence of iron affects both, the disorder level of the active phase and the oxidative charge around Ni during OER. The observed compositional, structural, and electronic properties of each system have been correlated with their electrochemical performance.

## Introduction

The reduction of anthropogenically generated CO_2_ by employing nonfossil fuel-based energy production has become a major focus on the international political agenda.^1^ High hopes have been put recently on hydrogen produced through water electrolysis using renewable energy sources, which could be used as an energy carrier, fuel, or feedstock.^2^ The oxygen evolution reaction (OER), the anodic half-reaction of water splitting, is a four-electron reaction process that requires a high overpotential.^3^ In this regard, it is believed that efficient and stable OER catalysts could help toward the large-scale implementation of water splitting.^4^ Up to date, the highest current densities are achieved with noble metal-based catalysts, such as Ir and Ru, in acidic media.^5,6^ In alkaline media, first-row 3d transition metals represent a non-noble metal-based alternative to be implemented in water electrolyzers.^7^ Among these, Fe-doped Ni-based catalysts stand out as highly promising,^8,9^ and they are already applied in liquid alkaline industrial electrolysis. Here, the role of iron boosting the catalyst activity of Ni-based electrocatalysts has been known since the 1980s and has attracted growing attention in the past decade.^10−12^ The complexity of studying the reaction arises from the transformation of the catalysts during OER to a working phase in the form of NiFe (oxy)hydroxide, as well as the intrinsic heterogeneity of nanoparticulate electrocatalysts.^13,14^

Several advances have been made in the understanding of the NiFe working catalyst in the last years with *operando* approaches, where working catalyst refers to the catalyst under operation conditions, i.e. inside the alkaline electrolyte and subjected to an applied potential. In the work from Dionigi et al., the active phase of a NiFe layered double hydroxide (LDH) was investigated with *operando* X-ray diffraction and X-ray absorption spectroscopy, where the relevance of the iron’s flexible electronic structure for the high reactivity was highlighted.^15^ In the same work, theoretical studies predicted that lattice oxygen plays a role in the reaction, which afterward was shown experimentally.^16^ However, there is still a lack of agreement in the literature regarding the effect that iron exerts on nickel. On the one hand, it has been described that iron assists in the oxidation of Ni^2+^, increasing the number of Ni^3+^ sites,^17^ and some works also claim that it promotes the formation and stabilization of Ni^4+^ species.^18^ Other studies claim that iron reduces the redox activity of Ni ions and increases the availability of Ni^2+^ species,^19−21^ which was linked to a kinetic competition between the metal oxidation reaction and the metal reduction reaction during oxygen release.^19^

Concerning the location of iron during the reaction in the Ni-based matrix, it has been proposed that it occupies octahedral sites of the Ni_1–*x*_Fe_*x*_OOH structure.^22^ Other researchers described a particularly active catalyst formed by a pure FeOOH linked to a NiOOH support.^23^ In a separate view, iron is suggested to partially dissolve and redeposit, with intercalation of iron also in the basal plane of the Ni matrix.^24^ Whether iron undergoes further oxidation or not during OER is another point of disagreement in the literature. Some studies suggest that iron remains stable as Fe^3+^.^25^ In contrast, other studies have discussed the presence of Fe^4+^.^26−28^ In the latter case, it has been emphasized that these oxidized species will be most active when they are situated at edges, corners, and defects.^28^ Regarding the identification of the active sites, it has been ascribed to either Ni sites,^18,21,29^ Fe sites,^22,24^ or dual Ni–Fe sites.^15,30^ The above-mentioned disagreements highlight the difficulties in getting universal insights into the working mechanisms of NiFe OER catalysts, and emphasize the need for further mechanistic studies on the structural and electronic transformation of the working catalyst.

A way to decrease the complexity level of the studies is by fully controlling the initial state of the precatalysts. This matter becomes even more crucial considering that aspects of the alkaline OER precatalysts such as surface orientation,^31,32^ thickness,^33^ morphology,^34^ iron concentration,^35^ and small variations in the sample preparation methods,^36^ also play a significant role in determining the activity toward OER. The use of crystalline oxides facilitates a greater degree of control over these systems. The proposed approach has been recently exemplified with Fe_3_O_4_ single crystals modified by Ni as well as with Co_3–*x*_Fe_*x*_O_4_ ordered thin films.^37,38^ In those studies, insights into the underlying mechanism of OER and accurate activity trends of the electrocatalysts were gained by combining surface science with electrochemical experiments. The single crystal approach has also been followed to study the influence of the NiO facet on the OER activity, where electrochemical studies on oriented thin films grown by magnetron sputtering revealed that, among the studied (111), (110), and (001) facets, the NiO(110) surface appears to form the most stable and active hydroxide.^31^

In the following work, we study the iron effect on ordered NiO thin films for the OER. Regarding iron and nickel-based catalysts in alkaline media, it is noteworthy to highlight that NiFe LDHs show a higher performance toward OER than NiFe oxides. However, oxides are better suited for industrial implementation.^2^ Thus, the study of the oxide precatalysts is of interest for applications in alkaline electrolyzers. The surface of the oxide precatalysts studied in this work will evolve to an oxyhydroxide phase at OER conditions,^11^ with the properties of this active surface skin layer being significantly influenced by the precatalyst’s structure and composition. We focused on two model systems grown under ultrahigh vacuum (UHV) conditions onto Pt single crystals: NiO(001) and Ni_0.75_Fe_0.25_O_*x*_(001). To analyze the modifications occurring on the as-prepared catalysts following an alkaline OER treatment, which consisted of a 2 h chronoamperometry at a potential where a current density of 1 mA/cm^2^ was reached, we employed a combination of surface-sensitive techniques. The catalysts were kept in an inert atmosphere when transferring them from the electrochemical cell to the main characterization setup, aiming to minimize the presence of contaminants. Given that only the catalyst’s first few nanometers at the surface actively engage in the reaction,^39^ the surface approach that we employ becomes significant in the investigation of these catalysts. We further study the thin films with *operando* Raman and grazing incidence X-ray absorption spectroscopy (GIXAS) to extract information on the active phase and electronic configuration of the working catalysts.

## Methods

### Sample Preparation

The thin films were grown in the preparation chamber of an ultrahigh vacuum (UHV) setup. The procedure to make NiO(001) thin films was inspired by a reported recipe to grow NiO(111) on Au single crystals and by a study focused on Ni single crystal oxidation.^40,41^ The NiO(001) and Ni_0.75_Fe_0.25_O_*x*_(001) thin films were prepared on Pt(001) single crystals (MaTeck GmbH, Germany). Pt, a harder noble metal than others like Au, was chosen as a substrate to grow the thin films. In this way, substrate bending issues that could affect the crystallinity of the oxide thin film overlayers could be avoided. No further substrates were studied for the growth of NiO(001). The Pt was cleaned by repetitive Ar^+^ sputtering and UHV annealing cycles, with an extra oxygen annealing step done after the first 2 cleaning cycles. Once a clear Pt(001) low electron energy diffraction (LEED) pattern was obtained, NiO was produced in repetitive cycles until 8 to 10 nm of Ni were deposited and oxidized. A cycle consists of two steps. The first step was to evaporate 2 to 4 nm of Ni at 625–650 K in a 1.7 × 10^–5^ to 2 × 10^–5^ mbar O_2_ atmosphere. Ni was evaporated with an e-beam-assisted triple evaporator (EFM 3T, Scienta Omicron) from a 99.9945% pure Nickel rod (Alfa Aesar). The evaporation rate of Ni was determined with a quartz microbalance and was around 0.5 Å/min. The second step consisted of annealing at 650 K for 10 min, using the same O_2_ pressure as for the Ni evaporation. In the case of Ni_0.75_Fe_0.25_O_*x*_(001), 1 to 2 Å of Fe were added at 525 K after the NiO(001) thin film was grown. Therefore, iron was evaporated after nickel, not simultaneously. Iron was 99.99+% pure (ChemPur), and the deposition rate was around 1.6 Å/min. After the iron addition, the thin film was annealed at 650 K in a 1.7 × 10^–5^ to 2 × 10^–5^ mbar O_2_ gas atmosphere for 10 min. This procedure was repeated until a 5.0% iron-to-nickel concentration was obtained, with a standard deviation across the catalysts of 0.8%. The concentration was calculated from X-ray photoelectron spectroscopy (XPS) data measured with an Al-K_α_ X-ray source.

### Characterization of the Thin Films

LEED, low energy ion scattering (LEIS), and XPS measurements were performed in the analysis chamber of the same UHV setup where the thin films were grown. XPS was measured with a Phoibos 150 (SPECS GmbH) analyzer and a monochromatized Al-K_α_ X-ray source. High-resolution spectra of the core level of interest were collected with a pass energy of 15 eV, except for the Ni and Fe 3p regions, which were measured with a pass energy of 60 eV. For the LEIS measurements, He ions with an energy of 800 eV were employed, with the same analyzer as the one used for XPS. The He pressure was set to 1 × 10^–7^ mbar, which led to an ion current of around 100 nA at the catalyst. All XPS and LEIS data have been analyzed with the software CasaXPS (Casa Software Ltd., Teignmouth, UK). The XPS binding energy scale has been calibrated employing the binding energy of NiO in Ni 2p_3/2_, 853.7 eV.^42^ A Shirley background was used to analyze the XPS spectra and a linear one for the LEIS spectra.

The catalysts were additionally characterized by atomic force microscopy (AFM), X-ray reflectivity (XRR), and synchrotron-based XPS. Both, AFM and XRR measurements were conducted in ambient air conditions. AFM measurements employed a Bruker Multimode 8 microscope in tapping mode. XRR data were collected using Bruker D8 Advance setups, utilizing a Cu source equipped with a Goebel mirror as well as a Lynxeye XE-T detector for the NiO(001) thin film measurement, and an EIGER2 R 250 K detector for the Ni_0.75_Fe_0.25_O_*x*_(001) thin film measurement. The XRR data were analyzed using the fit program FEWLAY,^43^ where two models were employed: one consisting of a NiO layer on Pt, and another one consisting of a Ni_0.75_Fe_0.25_O layer overlaying the NiO layer on Pt. The program uses a modified Parratt formalism taking into account the layer roughnesses as Gaussian-shaped fluctuations around the layer interface. The fit parameters included the layer thicknesses, the root-mean-square roughness of the surface and the interfaces, as well as the electron densities of the layers. For the electron densities, a closed layer with rock salt structure was assumed, with a lattice parameter upper limit of *a* = 0.4177 nm for NiO, and of *a* = 0.4213 nm for Ni_0.75_Fe_0.25_O. Synchrotron-based XPS and near edge X-ray absorption fine structure (NEXAFS) measurements were carried out at the Russian-German beamline (RGBL) at the BESSY II electron storage ring operated by the Helmholtz Centre for Materials and Energy, HZB.^44^ The catalysts were transferred to the beamline while exposed to air. Then, a cleaning step was performed to eliminate impurities and restore the LEED pattern of the thin films. This cleaning procedure involved heating the catalyst at 650 K in an O_2_ gas environment with a pressure of 5 × 10^–6^ mbar, lasting approximately 20 min.

### Electrochemical (EC) Setup and Protocol

For the electrochemical assessments, an EC cell was employed, connected to the load lock of the UHV chamber in which the thin films were both synthesized and analyzed. Detailed information regarding the cell and its cleaning process can be found in a previous work from our group.^38^ Essentially, this cell permits the transfer of the catalyst from the UHV environment to the electrolyte maintaining a completely inert atmosphere. In this way, it prevents the catalyst from being exposed to contaminants that may influence the catalytic activity and the subsequent measurements.

For the experiments, 0.1 M KOH electrolyte was prepared using semiconductor grade KOH pellets (99.99% trace metals basis, Sigma-Aldrich). The electrolyte was purified to ensure the removal of iron impurities following a chemical procedure described elsewhere.^45^ The reference electrode employed was an Ag/AgCl leakless electrode (EDAQ, 3.4 mol/L KCl), whose potential was calibrated with a reversible hydrogen electrode (RHE). A Pt mesh (MaTeck, 99.9%) served as the counter electrode.

The electrochemical protocol began with a cyclic voltammetry (CV) scan and anodic linear sweep voltammetry (LSV) where a geometric current density of 1 mA/cm^2^ was reached. Then, the potential was maintained for 2 h. Following the 2 h chronoamperometry (CA) step, a cathodic LSV was performed down to 0.04 V vs Ag/AgCl, and finally, the potential was swept up to the geometric current density of 1 mA/cm^2^. The scan rate for all the LSVs and CVs was set to 5 mV/s. To account for the ohmic drop, impedance measurements were conducted. Experiments were performed with an Autolab potentiostat (PGSTAT302N). Electrochemical impedance spectroscopy measurements were recorded from 10 kHz to 0.1 Hz, with an AC amplitude of 0.01 V_rms_, at the potential where a current of 1 mA/cm^2^ was reached.

### Operando Raman

*Operando* Raman measurements were performed in a Raman spectrometer (Renishaw, InVia Reflex) coupled with a confocal microscope (Leica Microsystems, DM2500M) equipped with a water immersion objective (Leica Microsystems, 63×, NA 0.9) and a motorized stage (Renishaw, MS300). A green laser (λ = 532 nm, Renishaw RL532) was used as the excitation source. The laser power was set between 0.5 and 5 mW to avoid laser damage on the thin films. The backscattered light was Rayleigh-filtered and collected in the 25–1290 cm^–1^ range with a 2400 lines mm^–1^ grating and directed to a CCD detector (Renishaw, Centrus). For the electrochemical measurements, a homemade cell made of PTFE equipped with a leakless Ag/AgCl electrode (Alvatek) and a Pt foil as the counter electrode was employed. A schematic of the cell can be found in Figure S1a. Fe-purified 0.1 M KOH was used as the electrolyte, the water immersion objective was isolated from the electrolyte by a Teflon film (DuPont, film thickness = 0.013 mm). The catalyst was transferred through air to the Raman cell. Before the Raman measurements, the selected potentials of interest were maintained for 5 min. This allowed for the stabilization of the electrochemical conditions before proceeding with the Raman measurements. The collection of each spectrum is the result of 15 s of exposure time and the accumulation of 5 to 10 sweeps.

### *Operando* Grazing Incidence X-ray Absorption Spectroscopy

GIXAS measurements were carried out at the KMC-2 beamline at BESSY II (HZB).^46^ Fluorescence mode was used with a Si-PIN photodiode detector. A Ni foil was measured on a separate detector in transmission mode for energy calibration purposes. The grazing incidence angle, determined by XRR measurements, was set to 0.2°, which is well below the critical angle of Ni at the Ni K-edge (0.315°) and results in surface-sensitive measurements. A homemade electrochemical cell was employed. A schematic of it can be found in Figure S1b. A Pt mesh was used as the counter electrode, and leakless Ag/AgCl as reference electrode. Fe-purified 0.1 M KOH was employed as electrolyte. The catalyst was transferred through air to the cell. Data normalization was conducted in the Athena software.^47^ The absorption edge positions of the Ni K-edge were calculated following a method described by Dau et al.^48,49^ Theoretical spectra were calculated using the FDMNES code.^50,51^ Details about the convolution parameters used as well as the applied shift can be found in the Supporting Information.

## Results

### OER Catalytic Performance

**Figure 1:**
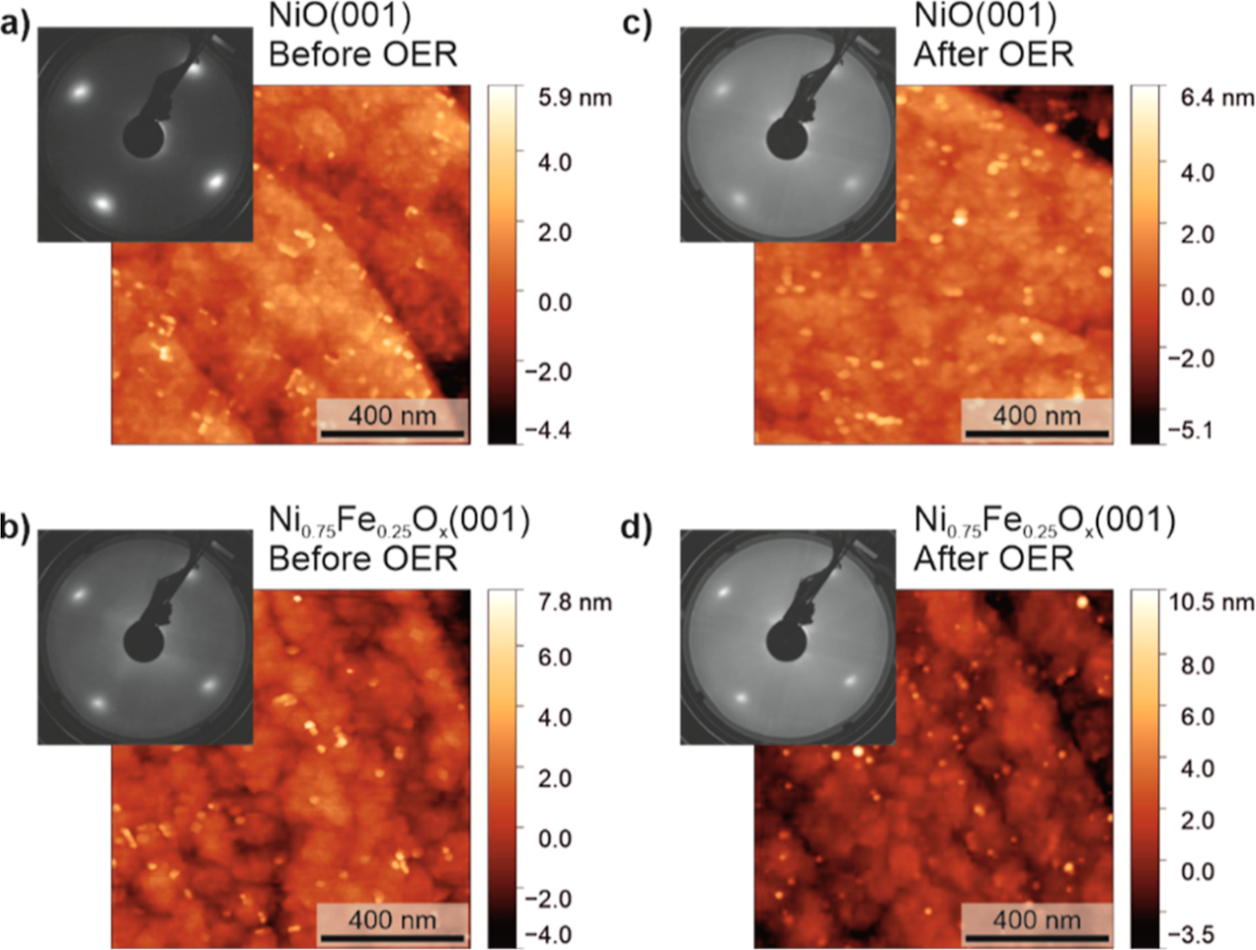
AFM and LEED images (65 eV) of NiO(001) (a,c) and Ni_0.75_Fe_0.25_O_*x*_(001) (b,d) acquired before (a,b) and after (c,d) a 2 h OER treatment in Fe-purified 0.1 M KOH.

Two thin film catalysts have been used in this work: NiO(001) and Fe-doped NiO(001). The LEED image of the as-prepared NiO(001) (Figure 1a) shows a cubic pattern, characteristic of the rock salt structure of NiO exposing the (001) surface. The LEED pattern of Ni_0.75_Fe_0.25_O_*x*_(001) (Figure 1b) shows the same surface structure as that of NiO(001), with an increased background and additional faint stripes. This demonstrates that the surface film ordering of NiO(001) has not changed due to iron addition, the structure is still the cubic rock salt one. The impact of a 2 h OER treatment on the surface structure is also illustrated in Figure 1. For the two electrocatalysts, after a 2 h OER treatment in Fe-purified 0.1 M KOH, an increase in the background is observed, indicating the growth of a noncrystalline phase on the surface. The intensity of the LEED pattern spot is slightly less damped in Ni_0.75_Fe_0.25_O_*x*_(001) than in NiO(001), suggesting that the noncrystalline phase formed during OER is thicker for the latter. Figure 1 also shows the AFM images that represent both catalysts’ surface morphology, as-prepared and after the 2 h OER treatment. The Ni_0.75_Fe_0.25_O_*x*_(001) thin film is slightly rougher than the NiO(001) thin film, with root-mean-square (rms) roughness values of 1.3 ± 0.2 nm and 1.2 ± 0.1 nm, respectively. The rms roughness values have been obtained from at least three 1 × 1 μm^2^ AFM images using the Gwyddion software. After the 2 h OER treatment, both catalysts’ morphology shows minimal changes, with rms roughness values of 1.2 ± 0.1 nm for NiO(001), and 1.3 ± 0.3 nm for Ni_0.75_Fe_0.25_O_*x*_(001). The scaling factors obtained from the AFM images analysis and used to calculate the electrochemical surface areas are listed in Table S1.

**Figure 2:**
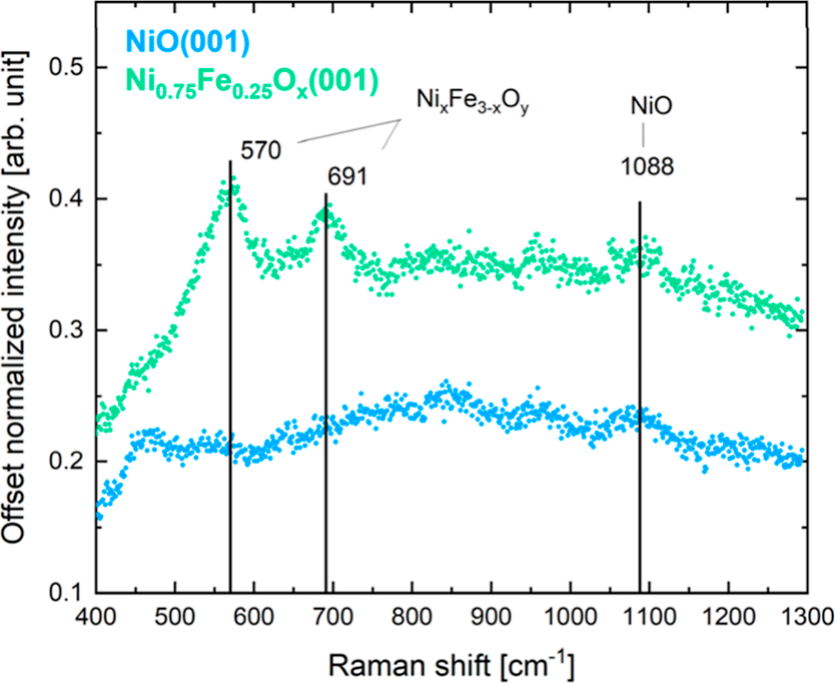
Raman spectra of NiO(001) and Ni_0.75_Fe_0.25_O_*x*_(001) thin films in air.

The initial state of the catalysts was further studied by Raman spectroscopy. The Raman spectra of the NiO(001) and the Ni_0.75_Fe_0.25_O_*x*_(001) thin films collected in air are shown in Figure 2. In the case of the NiO rock salt structure, first order phonon modes are forbidden, which should appear in the 300–600 cm^–1^ region.^52^ The weak features in that range observed for NiO can be attributed to defects in the structure. The peak at 1088 cm^–1^ is related to the second-order phonon mode 2LO of NiO.^52,53^ In the case of the Ni_0.75_Fe_0.25_O_*x*_(001) thin film, two new features are detected at 570 cm^–1^ and at 691 cm^–1^, respectively. Their presence has been recently related to having a mixed nickel and iron oxide in mainly rock salt structure,^54^ which also agrees with the LEED pattern of the system. The feature at 570 cm^–1^ has been identified as the longitudinal optical Raman scattering of a Ni–O vibration for a defective NiO, while the feature at around 691 cm^–1^ may be attributed to the presence of a spinel phase. The feature at 1088 cm^–1^ related to NiO is still visible for the Ni_0.75_Fe_0.25_O_*x*_(001) thin film. These results suggest that during the iron incorporation process, nickel ferrite-like structures are created, while the majority of NiO retains its original rock salt structure. To further validate that the majority of NiO stays in rock salt structure, Ni L edge of the as-prepared thin film oxides we measured with UHV NEXAFS. The Ni L edge spectra of both oxides, depicted in Figure S2, indicate that, in both cases, Ni atoms occupy octahedral positions. This confirms that the Ni_0.75_Fe_0.25_O_*x*_(001) thin film has kept the rock salt structure of the similarly epitaxially grown NiO.

**Figure 3:**
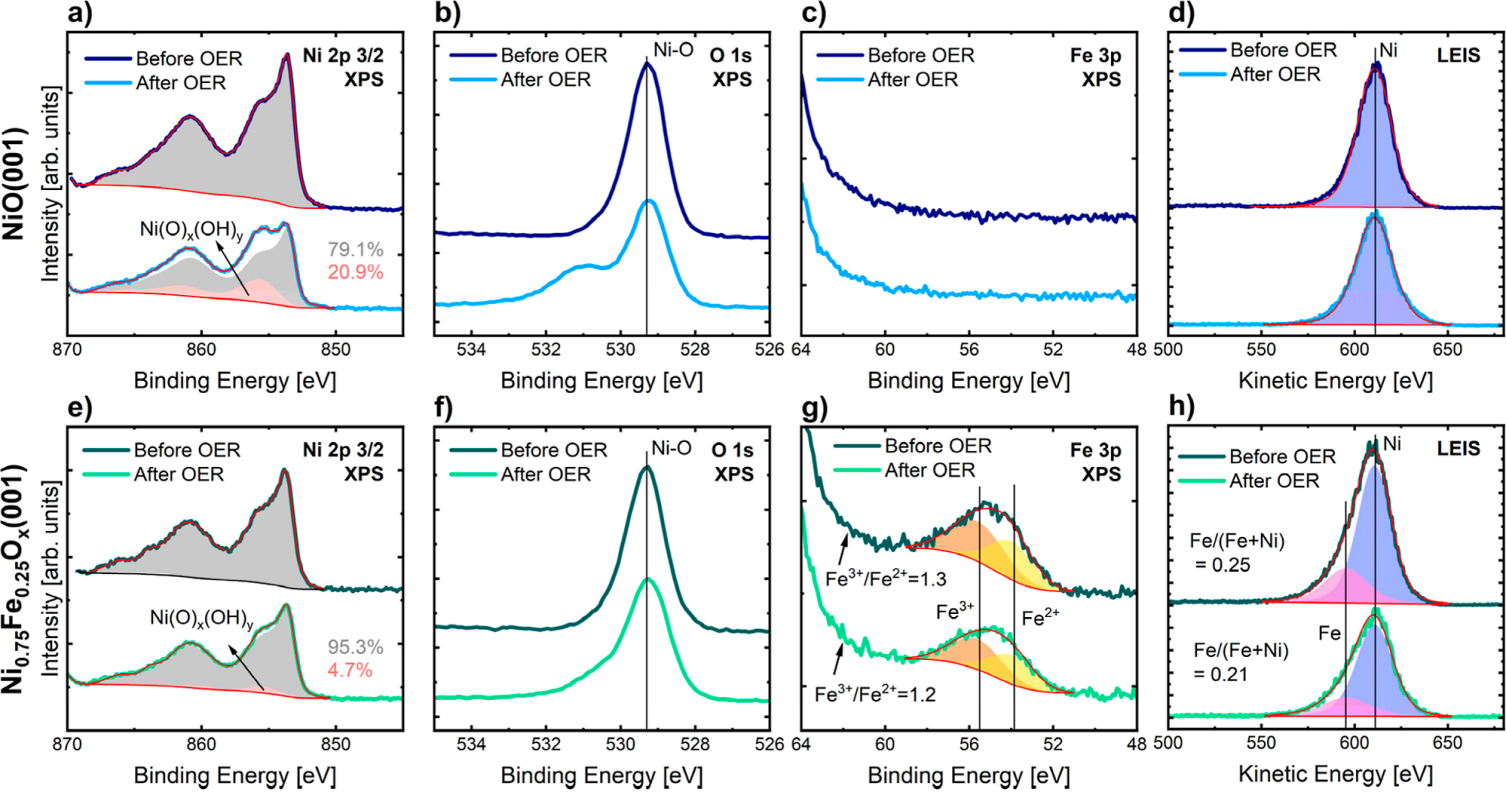
XPS spectra measured before and after a 2 h OER treatment in Fe-purified 0.1 M KOH of (a) Ni 2p_3/2_ of NiO(001), (b) O 1s of NiO(001), (c) Fe 3p of NiO(001), (e) Ni 2p_3/2_ of Ni_0.75_Fe_0.25_O_*x*_(001), (f) O 1s of Ni_0.75_Fe_0.25_O_*x*_(001), and (g) Fe 3p of Ni_0.75_Fe_0.25_O_*x*_(001). LEIS spectra from the Ni and Fe regions measured before and after a 2 h OER treatment in Fe-purified 0.1 M KOH of (d) NiO(001), and (h) Ni_0.75_Fe_0.25_O_*x*_(001). Background and fit profiles are represented with red lines.

Regarding the XPS data of the as-prepared NiO(001) (Figure 3a–c), the Ni 2p_3/2_ line shape corresponds to NiO,^42^ with features above 860 eV related to satellites, and the shoulder at high binding energy of the main line attributed to factors like multiplet splitting, nonlocal screening effects, and vacancies.^42,55,56^ Besides, there is only one peak in the O 1s spectrum at a binding energy of 529.3 eV corresponding to that of NiO,^57^ indicating that there is just one oxide species present in the NiO(001) catalyst. The absence of a peak in the Fe 3p region confirms that this thin film is free of iron impurities. The LEIS spectrum represented in Figure 3d shows the presence of surface Ni, which has a low-energy tail related to subsurface backscattering of the incoming ions that are reionized at the surface.^58^ Further, the Pt 4d XPS spectrum shows no presence of Pt (Figure S3), revealing that the thin film is closed. The closeness of the film is also supported by the fit results of the XRR data (see Table S2) shown in Figure S4a. The electron density profile obtained from the fit is displayed in Figure S4b. The fit moreover reveals a NiO film thickness of ca. 9.0 nm, confirming the nominal amount of NiO.

The XPS data of the as-prepared NiO catalyst doped with iron are shown in Figure 3e–g. The Ni 2p_3/2_ spectrum is similar to that of pure NiO, with a small increase in the shoulder at the high binding energy of the main peak. This effect can be linked to the presence of NiFe_2_O_4_, identified with Raman spectroscopy, and whose main peak in the Ni 2p_3/2_ XPS spectrum is located at 854.2 eV.^59^ In the O 1s spectrum, a predominant peak emerges, aligning with the NiO binding energy. There is a 0.05 eV increase in the full width at half-maximum of the O 1s peak compared to that of NiO(001). Additionally, the peak exhibits a higher asymmetry at the high binding energy range. These two effects can be explained by the contribution of the Fe–O bond, which has a binding energy in the 529.8–530.1 eV range.^57^ The Fe 3p XPS spectrum shows the contribution of both, Fe^3+^ and Fe^2+^. In the spectrum fitting, the binding energy of Fe^3+^ was set to 55.55 ± 0.05 eV, and that of Fe^2+^ to 53.85 ± 0.05 eV. A PseudoVoigt line shape with a 0.4 asymmetry factor was used. These parameters were based on a previous study.^60^ Here, it is worth mentioning that the analysis of the Fe 2p was hindered due to its overlap with the Ni LMM Auger peak when using the Al-K_α_ X-ray source. Regarding the two oxidation states of iron in the as-prepared Fe-doped NiO(001), the presence of Fe^3+^ can be attributed to the nickel ferrite. For Fe^2+^, we hypothesize that it is incorporated in the NiO rock salt structure, as the presence of a mixed oxide in the rock salt structure is also expected from Ni–Fe–O phase diagrams at the oxygen pressure employed to prepare the catalyst.^61^ Both these changes in the NiO structure would account for the faint stripes and the increased background observed in the LEED image presented in Figure 1b. The rise in the background may be related to the formation of an amorphous phase, while the faint stripes could be attributed to a distorted rock salt structure triggered by the addition of iron. Our observed structure also aligns with previous findings, which indicate that the formation of a fully inverse spinel structure, seen in NiFe_2_O_4_, and the loss of the NiO rock salt structure, only occurs at iron contents that are higher than what is present in our iron-doped thin films.^54,62^

The iron near-surface concentration and its depth profiling were determined by combining lab-based with synchrotron-based XPS measurements, where the photon energy of the X-rays was changed to perform the depth profiling. We use the areas corrected for the cross-section, asymmetry parameter, and photon flux of Ni and Fe at the 2p and 3p regions to quantify the amount of iron relative to the total amount of metals. The calculated concentrations of iron at several depths and the XPS spectra can be found in Figure S5. The figure reveals the existence of an iron concentration gradient in the film with the highest concentration at the surface. This is most probably related to the method used to grow the iron-doped thin film, where iron was added onto the surface of nickel oxide. The iron concentration measured when utilizing the less surface-sensitive XPS measurement conditions is 5%, corresponding to electrons with an inelastic mean free path of (IMFP) 22.8 Å. In contrast, the most surface-sensitive measurement reveals an iron concentration of 22%, where electrons have an IMFP of 5.9 Å. It is plausible that an even more elevated content of iron may be present at the outermost surface of the thin film. To assess this outermost surface iron concentration, we employed LEIS data analysis due to its solely surface specificity. The line shape and kinetic energy of the NiO(001) LEIS spectrum were used as the reference of the Ni contribution when fitting the LEIS spectrum of the thin film doped with iron. From the peak areas of the LEIS spectra illustrated in Figure 3h, we found that the iron concentration at the surface is 25%. For this reason, the Fe-doped NiO thin film is referred to as Ni_0.75_Fe_0.25_O_*x*_(001) in this work.

The XRR data of Ni_0.75_Fe_0.25_O_*x*_(001) has been fitted with two models: a NiO/Pt single-layer model and a Ni_0.75_Fe_0.25_O/NiO/Pt double-layer model. The fitted data and the electron density profile obtained from each fit are depicted in Figure S4c–f, and the fit results are summarized in Tables S3 and S4. Due to the similarity in the electron density of iron and nickel, finding a good fit with the single-layer model suggests that iron is mixed in the NiO(001) thin film. The double-layer model also shows a good fit, indicating that the XRR data is compatible with having most iron incorporated on the surface of the NiO thin film. The fit results of the bilayer model suggest that the thickness of the NiO layer is ca. 10.0 nm, whereas that of the Ni_0.75_Fe_0.25_O overlayer is ca. 0.9 nm. Additionally, both models show that the Ni_0.75_Fe_0.25_O_*x*_(001) thin film has a rougher surface compared to NiO(001), as found by AFM data analysis.

Figure 3 also illustrates the impact of the 2 h OER treatment on the composition of the thin film surfaces. Deconvoluting the Ni 2p_3/2_ spectra after the reaction without air exposure is a complex task due to the overlapping spectra of the various possible species that can be formed, such as α-Ni(OH)_2_, β-Ni(OH)_2_, β-NiOOH, γ-NiOOH, and layered double hydroxides.^57,63^ To overcome this issue, lineshapes of the as-prepared thin films were generated using CasaXPS. Then, the spectra collected after OER were fitted using those lineshapes and an additional one, which has been denoted as Ni(O)_*x*_(OH)_*y*_. The main peak of the line shape after OER lies at a binding energy of 855.6 eV for NiO(001), and 855.4 eV for Ni_0.75_Fe_0.25_O_*x*_(001). These binding energies are between the reported main peak binding energies of Ni(OH)_2_ (855.3 eV) and NiOOH (855.8 eV).^64^ A comparison of Figure 3a,e reveals that the conversion of the NiO during the reaction is higher in the case of NiO(001), where the Ni(O)_*x*_(OH)_*y*_ line shape area accounts for 20.9% of the total area. In contrast, for Ni_0.75_Fe_0.25_O_*x*_(001), those species represent 4.7% of the total area of Ni 2p_3/2_. The estimated thickness of the Ni(O)_*x*_(OH)_*y*_ layer calculated from the Ni 2p_3/2_ XPS data and assuming a flat layer accounts for around one monolayer of Ni(OH)_2_, ca. 4 Å, in the case of NiO(001), and less than a monolayer in the case of Ni_0.75_Fe_0.25_O_*x*_(001). The formation of (oxy)hydroxide species during the reaction, that remain stable on the surface after removing the applied potential, can also be deduced from the O 1s spectra presented in Figure 3b,f, as there is an increase in the spectral contributions in the binding energy range associated with –(OH)_2_ (530.9–531.3 eV), NiOO_1–*x*_–OH (530 eV), NiOOH (530.65 eV), NiOOH (531.88 eV), and FeOOH (531.2–531.4 eV).^57,65,66^ All of these binding energies are higher than the O 1s main peak of NiO(001) and Ni_0.75_Fe_0.25_O_*x*_(001) (529.3 eV), allowing us to identify the contribution of the (oxy)hydroxide phase relative to that of the oxide. However, distinguishing between the hydroxide and oxyhydroxide phases themselves remains complex with the experimental tools at hand. The increase is more pronounced in the case of NiO(001), leading to the same observation as that obtained from the Ni 2p_3/2_ spectra: more oxide is transformed into (oxy)hydroxide at the surface of the catalyst without iron. Thus, XPS data analysis reveals that both catalysts react differently to the OER conditions: NiO(001) suffers a higher conversion toward an (oxy)hydroxide phase than Ni_0.75_Fe_0.25_O_*x*_(001). The XPS spectra of the Fe 3p region shown in Figure 3c provide evidence that NiO(001) is free of contamination from iron impurities after the reaction. In Ni_0.75_Fe_0.25_O_*x*_(001), the analysis of the Fe 3p XPS region (Figure 3g) shows that the Fe^3+^ to Fe^2+^ ratio decreases slightly after the OER treatment. Besides, a reduction in the iron concentration has been confirmed by the analysis of the LEIS spectra (Figure 3h), which indicates that iron content in the catalyst is reduced from 25 to 21%.

**Figure 4:**
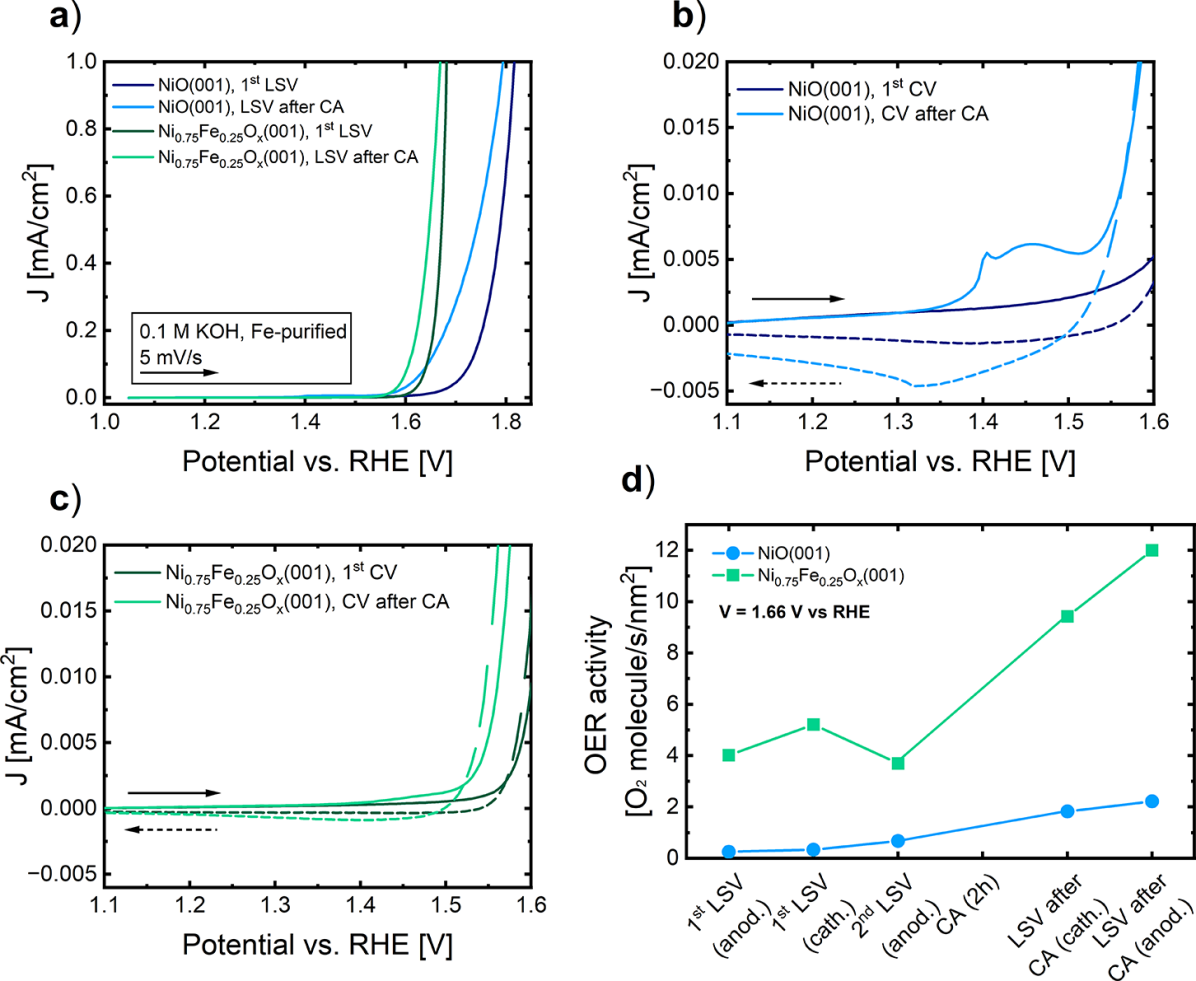
(a) Anodic LSV curves of NiO(001) and Ni_0.75_Fe_0.25_O_*x*_(001) before and after a 2 h OER treatment. (b) Nickel redox reaction area for NiO(001) and (c) Ni_0.75_Fe_0.25_O_*x*_(001) before and after a 2 h OER treatment. (d) Oxygen evolution activities at 1.66 V vs RHE at different states of the EC experiment for NiO(001) and Ni_0.75_Fe_0.25_O_*x*_(001), where “anod.” means anodic, and “cath.” means cathodic, related to the direction of the LSV scans.

To understand the effect of iron on the electrocatalytic performance of NiO(001), an initial CV followed by an anodic LSV were measured until reaching a potential that corresponds to a current density of 1 mA/cm^2^. At this potential, the OER reaction was left running to produce a 2 h chronoamperometry (CA). After that, a cathodic LSV and an anodic one were recorded to study the effect of the OER conditioning. Figure 4 shows the anodic LSVs before and after the OER treatment (Figure 4a–c), and the OER activity at different stages of the EC experiments (Figure 4d). The current densities and the activities shown in the figure have been normalized using the surface area scaling factor calculated from the AFM images (Table S1). We note that the surface area normalized current of the planar NiO thin films, 1 mA/cm_oxide_^2^, reflects a highly relevant regime of reaction conditions equivalent to 200 mA/cm_geometric_^2^ achieved for NiO powder catalysts, assuming a specific surface area of 100 m^2^/g and a typical loading of 0.2 mg/cm^2^.^67^ The employed OER protocol also allows us to study the activity of the thin films without the interference of the Pt, as the thin films stay closed after the EC (Figure S3). When conditioning the NiO thin film by consecutive CV cycles, which is an activation method that has a larger impact on the initial precatalyst than CA,^68^ Pt was detected by XPS after the activation (Figure S6). This detection of Pt prevents us from estimating the activity of the thin film accurately, as the contribution of Pt must be considered. For this reason, we focused our experiments and discussions on the results attained after a 2 h CA activation process.

The comparison of the anodic LSV scans shown in Figure 4a indicates that the OER onset potential of the NiO(001) catalyst decreases in the presence of iron, and also that this potential is reduced after the 2 h OER treatment for the two systems. The nickel redox reaction area before and after the 2 h OER treatment for the two systems, depicted in Figure 4b,c, corresponds to the oxidation of Ni^2+^, and is usually attributed to the oxidation of Ni(OH)_2_ to NiOOH.^45^ A clear redox feature is observed for the NiO(001) catalysts after the OER treatment, indicating that NiOOH has been created at OER conditions. A weaker Ni redox feature is observed for the Ni_0.75_Fe_0.25_O_*x*_(001) thin film. The comparison of the redox area after the OER treatment for the two systems, calculated from the cathodic LSV highlighted area in Figure S7, indicates that the redox feature is suppressed by around 64% in the case of Ni_0.75_Fe_0.25_O_*x*_(001). This suppression cannot be explained just by the decrease in the number of Ni atoms accessible on the surface, as that should account for a suppression of 25%. Thus, the addition of iron reduces the redox activity of nickel atoms during OER, an effect previously discussed,^19^ and in agreement with the observations derived from the XPS data analysis. This effect was also previously discussed by Görlin et al.,^19^ who observed that it occurs regardless of whether the catalyst is supported or not, indicating that iron’s effect on Ni is independent of the support used. Additionally, the same effect of a decrease in the redox activity was observed using randomly oriented Ni_1–*x*_Fe_*x*_O_*y*_ nanoparticles,^20^ suggesting that the results obtained for our epitaxial NiO(001) thin films can be extrapolated to more realistic systems. As previously noted, our NiFe oxide precatalysts exhibit a gradient in the iron composition, with the highest concentration of Fe at the surface. Earlier studies also observed a decrease in Ni redox activity with precatalysts that have this type of iron gradient,^20,69^ suggesting a potential influence of the iron gradient on Ni redox activity that could be worth investigating in a separate work.

Figure 4d displays the OER activity data corresponding to 1.66 V vs RHE at different stages of the electrochemical experiments. All the LSVs used to obtain these activity data are shown in Figure S8. At all the stages of the experiment, the catalyst that contains iron generates more oxygen. If we observe the cathodic LSV after the CA, one can see that the activity of both catalysts has increased during the OER treatment. This increase in the activity can be attributed to the formation of the oxyhydroxide phase during OER, a phase that will cover the surface of the catalysts creating a thin skin layer more active than the oxide itself. However, in the case of the catalysts doped with iron, the decrease in the iron-to-nickel ratio following the EC experiment, as revealed by the LEIS spectra analysis, suggests that some iron dissolves during the OER treatment. Thus, both the formation of the oxyhydroxide phase and the dissolution of some iron could play a role in enhancing the activity of the catalyst doped with iron. Interestingly, despite the greater formation of oxyhydroxide observed in NiO(001), the catalyst activity remains lower, indicating that the created oxyhydroxides in the two catalysts have distinct properties.

With the aim of checking how the ordered NiO thin film behaves relative to a disordered one, we have compared the electrochemical response of our epitaxial NiO(001) thin film with a disordered NiO thin film. Further details on these investigations and comparison can be found in the Supporting Information Section and Figures S9–S11.

### Active Phase during OER

The data indicate that the presence of iron increases the OER activity of NiO(001) and reduces the thickness of the Ni(O)_*x*_(OH)_*y*_ layer formed on the oxide surface. These observations have been derived from the analysis of the XPS spectra and electrochemical experiments. All the characterizations were conducted in an inert atmosphere, providing valuable information on how the reaction affects the as-prepared catalysts. However, these experiments do not represent the real working catalyst, since the spectroscopic characterization took place before and after OER, despite the lack of air exposure of the catalysts during the transfer. Thus, further *operando* resonance Raman experiments were performed to gain insight into this matter. The Raman intensity of the two vibrations of NiOOH is enhanced by resonance effects which makes it possible to detect them even for very thin layers.^70,71^

**Figure 5:**
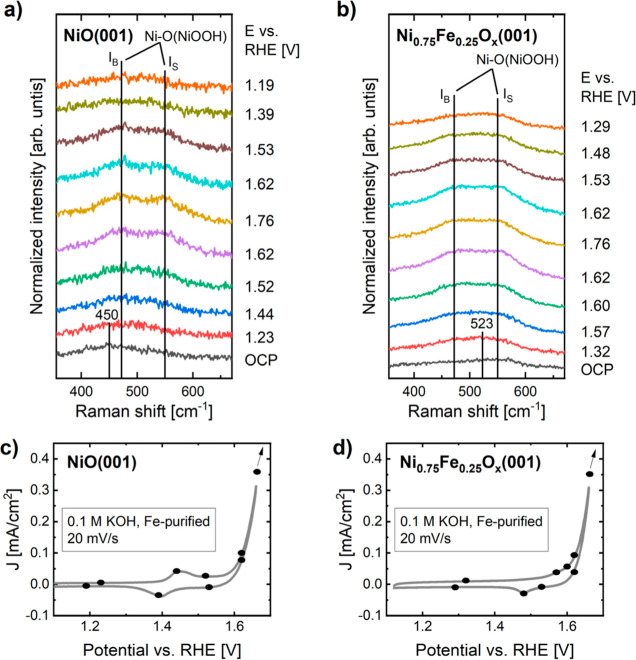
*Operando* Raman spectroscopic measurements of (a) NiO(001) and (b) Ni_0.75_Fe_0.25_O_*x*_(001), and the corresponding CV curves of (c) NiO(001) and (d) Ni_0.75_Fe_0.25_O_*x*_(001). Dots of the CVs indicate the potentials at which Raman spectra were collected.

Figure 5 illustrates the evolution of the Raman spectra obtained during electrochemical experiments, and their corresponding CVs. For the NiO(001) thin film under open circuit potential (OCP) conditions, the presence of a wide peak centered around 450 cm^–1^ indicates that Ni(OH)_2_ has been formed.^72^ The coexistence of both NiO and Ni(OH)_2_ at 0.1 M KOH is anticipated based on previous reports.^73^ When the potential is increased, two peaks appear in the spectra at around 470 and 550 cm^–1^. They correspond to the bending and stretching vibrational modes of the Ni–O bonds in NiOOH, respectively,^71,74−76^ and have been labeled as *I*_B_ and *I*_S_ in Figure 5. Their presence indicates that NiOOH was formed. Further evidence can be observed when the potential is decreased below the redox feature of the CV, resulting in a decrease in the intensity of the two peaks as a consequence of the back-transformation of NiOOH into Ni(OH)_2_. This behavior can also be seen in Figure S12, where the spectra are displayed after subtracting from the working data the spectra measured at a potential preceding the redox feature of the CVs. This subtraction enables us to observe more clearly the formation and disappearance of NiOOH. For the Ni_0.75_Fe_0.25_O_*x*_(001) thin film, the Ni(OH)_2_ feature centered around 450 cm^–1^ is not observed at OCP conditions. However, when applying a potential below the redox peak (1.32 V vs RHE), a feature centered at 523 cm^–1^ is detected, corresponding to NiFe LDH.^77,78^ When increasing the potential, the *I*_B_ and *I*_S_ vibration modes of NiOOH are visible. The decrease in the intensity of those two peaks also follows the redox feature of Figure 5d, particularly visible in Figure S12, further confirming that they are related to the oxidation of nickel. A difference between the NiOOH formed on both samples has been observed when comparing the *I*_B_/*I*_S_ ratio calculated from the spectra in Figure 5a,b. The mean *I*_B_/*I*_S_ ratio for the NiOOH grown on NiO(001) thin film is 1.3 ± 0.2, whereas for NiOOH grown on Ni_0.75_Fe_0.25_O_*x*_(001), it is 1.02 ± 0.04. A decrease in the *I*_B_/*I*_S_ ratio is an indication of an increase in the disorder level of NiOOH, attributed to defects and disorder in the lattice, which have been linked to the incorporation of iron into NiOOH.^35,72,74,77,79^ With this information, we can conclude that the NiOOH grown on Ni_0.75_Fe_0.25_O_*x*_(001) has a higher structural disorder level, probably due to the formation of an iron doped NiOOH phase.

### Effect of Iron Addition on Ni Oxidative Charge during OER

To further evaluate the catalysts under working conditions, *operando* GIXAS experiments were conducted. By measuring at a grazing incidence angle, the ratio of the signal from the catalyst’s surface relative to that of the bulk and the background is increased, allowing us to track the changes occurring in the area of the catalyst nearest to the surface.

**Figure 6:**
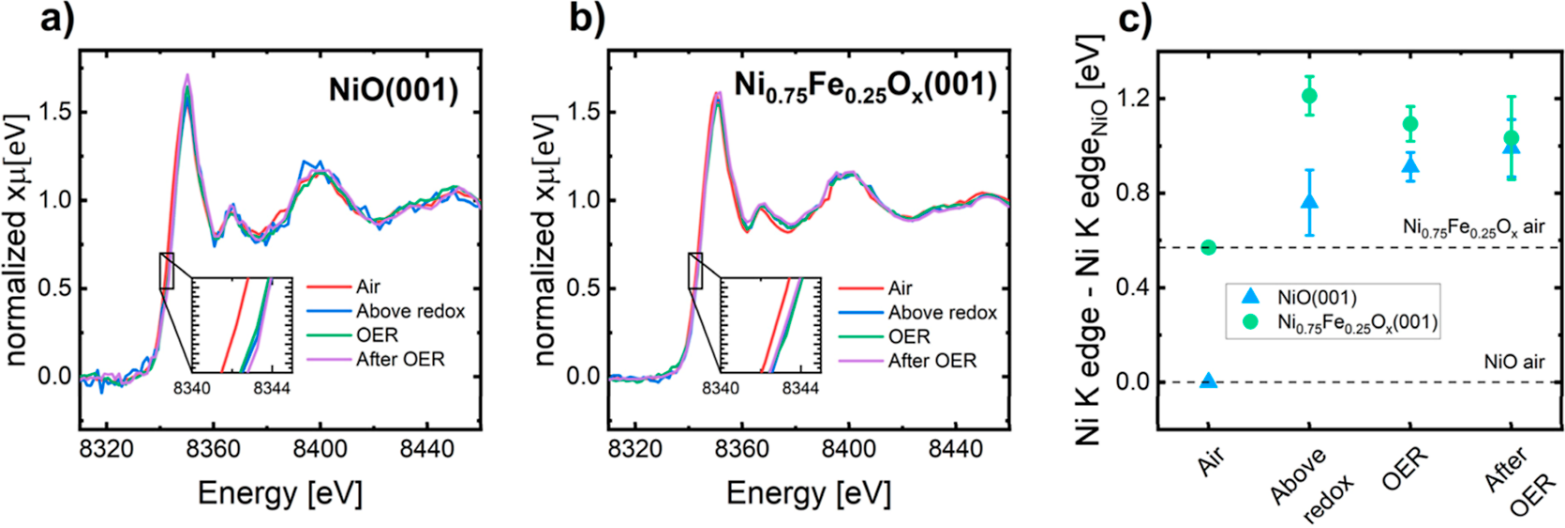
*Operando* GIXAS spectra measured at several conditions for (a) NiO(001) and (b) Ni_0.75_Fe_0.25_O_*x*_(001). (c) Ni K-edge position shifts relative to the Ni K-edge position of NiO thin film in air. The standard deviations of the individual spectra are shown as error bars.

NiO(001) and Ni_0.75_Fe_0.25_O_*x*_(001) thin film catalysts were analyzed under four conditions. First, in ambient air. Then, at a potential above the redox feature associated with the oxidation of nickel and before the OER onset potential (named “above redox”). After that, under OER conditions, and finally, after OER conditions at a potential below the redox feature (named “after OER”). The measured potentials at each condition are indicated in the Supporting Information, and the normalized GIXAS spectra are shown in Figure 6a,b. The variation of the absorption edge position of the Ni K-edge is shown in Figure 6c. Interestingly, the oxidation state of the nickel ions in the two pristine catalysts is not the same: there is a positive shift of 0.57 eV from NiO(001) to Ni_0.75_Fe_0.25_O_*x*_(001), indicating that the Ni initially present in Ni_0.75_Fe_0.25_O_*x*_(001) is more oxidized. Previous studies have reported the same effect of iron on Ni at resting conditions.^18,20^ When transitioning to the potential above the redox feature, the shift in the Ni K-edge position of Ni_0.75_Fe_0.25_O_*x*_(001), in comparison to its state in air, is 0.65 eV, which is smaller than the shift observed for NiO(001), 0.92 eV. This difference aligns with the previously observed behavior of a higher Ni redox activity in the iron-free catalyst. When increasing the potential up to OER conditions, the edge position of the X-ray absorption near edge structure (XANES) spectrum for Ni(001) increases, whereas that of Ni_0.75_Fe_0.25_O_*x*_(001) does not. Upon returning to a potential preceding the redox feature, no reduction of the nickel has been detected, possibly attributed to a higher reduction time needed for some of the oxyhydroxides. A similar behavior has been previously reported in other studies.^80,81^

To extract information about the active phase formed during the reaction, we employed the Δμ technique, which isolates the weak changes in the XANES spectra by a differential method.^82^ Herein, we used the NiO(001) XANES spectrum acquired in air as a reference. To correlate the changes with some specific compounds, the theoretical XANES spectra of NiO and plausible species that could be formed have been calculated: α-Ni(OH)_2_, β-Ni(OH)_2_, β-NiOOH, and γ-NiOOH. Even though it is known that the presence of iron will modify the structures of those species to some extent,^15^ our focus is on the pure phases due to their well-known structures. The theoretical spectra and their corresponding Δμ profiles are shown in Figures S13 and S14. Comparing the experimental and the theoretical Δμ XANES spectra depicted in Figure S14 evidence that in both systems the Δμ features resemble those of the NiOOH species, which aligns with the conclusions from the *operando* Raman experiments. When decreasing the potential to the last condition measured, the Δμ features suggest that the NiOOH phase is still present in the catalyst, further indicating that the active phase has not yet been reduced.

## Discussion

Under alkaline OER conditions, NiO(001) and Ni_0.75_Fe_0.25_O_*x*_(001) catalysts are covered, as expected, by a thin skin layer in the form of an oxyhydroxide phase. This skin layer has been detected by XPS analysis, *operando* Raman, and *operando* GIXAS spectroscopy measurements. The formation of the oxyhydroxide skin layer positively impacts the OER activity of the catalysts: after a 2 h OER treatment, the activity increases in both systems (Figure 4d). This oxyhydroxide phase is considered to be the active phase of the oxides.

Doping the NiO(001) thin film catalyst with 25% iron enhances its catalytic activity toward OER, both before and after an alkaline OER treatment. We found that iron has the following two effects in the oxyhydroxide phase. The first one is related to its thickness: Fe limits the growth of the oxyhydroxide phase. This effect has been concluded from the XPS data analysis, where we have detected that, after a 2 h OER treatment, ca. 20% of Ni is in the form of a Ni(O)_*x*_(OH)_*y*_ for the NiO(001) catalysts (Figure 3a), whereas only ca. 5% of Ni has been converted to Ni(O)_*x*_(OH)_*y*_ for the NiO(001) film doped with iron (Figure 3e). Besides, a smaller shoulder in the high binding energy area of the O 1s peak after the OER treatment for the sample doped with iron (Figure 3b,f) also indicates that less (oxy)hydroxide has been formed in the presence of iron. The same effect has been concluded when comparing the Ni redox features visible after the OER treatment, represented in Figure 4b,c: the area of the Ni redox feature is suppressed by ca. 64% after the 2 h OER treatment in the presence of iron. Another indication of this effect of iron can be inferred from the GI-XAS spectroscopy data analysis, as we observed a smaller shift in the Ni K-edge position of Ni_0.75_Fe_0.25_O_*x*_(001) than in NiO(001) when increasing the applied potential on the catalysts. This phenomenon has also been observed in previous studies during alkaline OER.^19,20,83^ In general, we have to note that we identified a qualitative agreement between the near-surface adaptation to the reaction conditions and the irreversible hydroxylation of the near-surface sites. Here, we refer to hydroxylated sites not being converted back to oxide sites when removing the applied OER potential as irreversible hydroxylation. The second effect that iron exerts in NiOOH is related to its structural disorder: *operando* Raman data show that the NiOOH grown on Ni_0.75_Fe_0.25_O_*x*_(001) presents a higher level of disorder than that grown on NiO(001), probably as a consequence of incorporating Fe in the NiOOH phase, as previously noted for these catalysts.^35,79^ Thus, Fe addition to the NiO thin films hinders the irreversible formation of NiO_*x*_(OH)_*y*_ under OER conditions and disorders its structure, avoiding the formation of a more ordered bulk-like NiOOH phase. These observations indicate that an increase in the disorder level of NiOOH is an important effect to consider when designing a Ni-based catalyst for alkaline OER. However, based on the conclusions from our work, the increase in activity brought about by the Fe addition cannot be solely attributed to the higher structural disorder induced on NiOOH. It is likely that the higher disorder, which has been reported as responsible for the apparent increase in activity of Co_3_O_4_ in Fe-containing Co_3_O_4_,^84^ is not the only factor contributing to the increased activity, but that electronic changes induced in Ni oxide by Fe upon its incorporation might also play a significant role.

For the case of the catalyst doped with iron, the analysis of LEIS data shown in Figure 3h revealed that, after the CA, the composition of the surface oxide has changed from Ni_0.75_Fe_0.25_O_*x*_ to Ni_0.79_Fe_0.21_O_*x*_. This decrease in iron content indicates a dissolution of iron during OER, which has also been observed in previous works.^24^ Then, the increase in the activity of the catalysts after the OER treatment could be a combination of the following: the formation of very thin (<0.4 nm) and disordered Fe-doped NiOOH, and the dissolution of iron to reach a more optimum surface composition. In the as-prepared Ni_0.75_Fe_0.25_O_*x*_(001) catalysts, two Fe states, Fe^2+^, and Fe^3+^, have been identified, where Fe^3+^ ions form NiFe_2_O_4_, and Fe^2+^ ions are most likely integrated into the NiO rock salt matrix. XPS data revealed a decrease in the Fe^3+^ to Fe^2+^ ratio after the OER treatment (Figure 3g). This reduction might indicate that the iron dissolved initially existed in the 3+ oxidation state, which could be associated with the dynamic involvement of Fe^3+^ in the reaction, while Fe^2+^ could have a lesser role. This observation could be linked to some previous works that identified Fe^3+^ centers in the active phase of NiFe catalysts at OER conditions.^19,22^ However, further *operando* evidence will be needed to confirm this assertion.

Regarding the results of the *operando* GIXAS experiments, we observed that in the case of NiO(001), the oxidative charge of Ni increases when transitioning to the OER condition. However, the opposite is true for Ni_0.75_Fe_0.25_O_*x*_(001). The increase in the oxidative charge in NiO(001) could be linked to a greater accumulation of the NiOOH phase on its surface, as inferred from the XPS results, or to an increase in the oxidation state of the nickel atoms in NiOOH from Ni^3+^ to Ni^4+^. The latter is however highly unlikely due to the instability of the Ni^4+^ species. Besides, there is another parameter that would also lead to an apparent increase of the oxidative charge in the presence of O ligands within a reactive environment in the near-surface electronic structure of Ni^3+^, namely, the formation of nickel-oxyl, Ni^III^–O^•^. In fact, in the case of Co^3+^, which also accumulates oxidative charge during the OER,^84^ it has recently been found that the increase in the oxidative charge is more likely linked to the formation of oxyl-species and charge reorganization in the 3d orbital, rather than to an increase in the oxidation state to Co^4+^.^49^ A similar effect was also described for Ir, where the oxyl species were even more stable.^85−89^ In light of the latter studies, we believe that both, the accumulation of NiOOH and the formation of oxyl-species are responsible for the increase in the Ni K-edge position observed for NiO(001). Further studies about the electronic configuration of oxygen could help to disentangle the reasoning behind the higher apparent oxidative charge of NiO(001), as the ones from the recent study of Wartner et al.^90^ Regarding the Ni_0.75_Fe_0.25_O_*x*_(001) catalyst, the lack of an increase in the Ni K-edge position when transitioning to OER conditions may be related to the faster generation of O_2_ in Ni–Fe sites, which will bypass the nickel oxidation reaction to create NiOOH and reduce the accumulated oxyl species on the surface. In a previous work, it was also proposed that the faster generation of O_2_ due to iron incorporation into the Ni catalysts prevents the accumulation of oxidized Ni species at OER conditions.^25^ This conclusion was drawn using various methods, including XAS at OER conditions. With our grazing incidence *operando* approach, we were able to enhance the limited surface sensitivity of this method, and to analyze how the Ni K-edge position shifts from above-redox conditions (prior to the OER onset) to OER conditions. The trend observed, where the Ni oxidative charge does not increase when transitioning to OER conditions in the presence of iron, further supports the idea that the higher activity of the NiFe oxide prevents the accumulation of highly oxidized Ni–O species or that their lifetime is substantially shorter due to a faster catalytic turnover. These findings imply that the active centers could be Ni/Fe pairs, aligning with the results of some of the previous works.^15,25^

Interestingly, previous studies reported that the shift of the Ni K-edge position toward higher energies at OER conditions is larger in the absence of iron.^20,25,91^ Some of these studies proposed that this phenomenon can be attributed to a higher stability of the oxidation state of Ni in the presence of iron.^20,25^ This observation does not contradict our results, as we also observe that the difference in the Ni K-edge XANES spectrum at OER with respect to the XANES spectrum of the catalyst in air is smaller for the system that contains iron. Besides, there may be subtle differences in the initial, as-prepared catalysts. As previously stated, in our case the Ni K-edge position exhibits a positive shift of +0.57 eV in the presence of iron, whereas previous studies reported a shift of only +0.2 eV,^20^ indicating that the catalysts under investigation are not necessarily comparable. The catalyst preparation method, the precise iron concentration, and the purity of the electrolyte employed are also parameters that differ between studies, which could account for variations in the active phase. These results highlight the importance of properly controlling and understanding the initial state of the precatalyst in order to compare activities and effects between studies, since such state will determine the transformations that it will experience during operation.

## Conclusions

In this work we studied the OER electrocatalytic performance of two model thin film catalysts with identical surface orientation, NiO(001) and Ni_0.75_Fe_0.25_O_*x*_(001), focusing on the effect of iron on the electrocatalytic performance. Our in-depth surface science characterization of these materials confirmed that the intrinsic activity of Ni_0.75_Fe_0.25_O_*x*_(001) is higher than that of NiO(001). Additionally, a 2 h OER treatment was found to activate both catalysts. *Operando* spectroscopy measurements show that oxyhydroxide is present under reaction conditions in both systems, with distinct characteristics depending on the precatalyst. In the NiFe oxide, a thinner oxyhydroxide overlayer forms, which additionally has a higher level of structural disorder, probably related to the incorporation of Fe in NiOOH. Some iron loss by dissolution at OER conditions was observed for the Ni_0.75_Fe_0.25_O_*x*_(001) precatalyst. We attribute the increase in the activity of this precatalyst after the OER treatment to both, the formation of disordered Fe-doped NiOOH, and the creation of a more active surface composition by Fe dissolution, leading to a Ni/Fe ratio of 0.79/0.21. By conducting *operando* grazing incidence XAS experiments, we identified that the oxidative charge of Ni present in NiO(001) increases under OER conditions, but this effect was not observed for Ni_0.75_Fe_0.25_O_*x*_(001), which has been linked to its higher activity toward OER.

In summary, our results evidence several effects of iron when doped to NiO. It constrains the growth of the oxyhydroxide phase, increases its structural disorder, and modifies the electronic charge around Ni during OER, likely due to the faster oxygen generation of Ni–Fe reaction centers. Our findings further emphasize the significance of understanding the evolution of pristine catalysts during reaction, which is affected by the characteristics of the as prepared state, and highlight the utility of model thin films and surface-sensitive approaches for the detailed investigation of electrochemical processes.

## Supporting Information Available

Sketches of the *operando* cells, Ni L edge spectra, XPS Pt spectra, XRR measurements data, depth profiles of the NiFe system, 100 CVs on NiO(001), LSVs before and after OER, OER on a disordered NiO thin film catalyst, *operando* Raman subtracted spectra, simulated XANES spectra, Δμ XANES spectra, potentials measured during *operando* GIXAS, notes about Ni K-edge simulations, surface area scaling factors, XRR fit parameters (PDF)
