# Supplementary material for: Effect of Iron Doping in Ordered Nickel Oxide Thin Film Catalyst for the Oxygen Evolution Reaction
Source: ACS Catal. 2024 Sep 11;14(18):14219–32. doi: 10.1021/acscatal.4c02572 (PMC11421220; doi:10.1021/acscatal.4c02572)
Supplement: Supplementary file 1 — cs4c02572_si_001.pdf [file cs4c02572_si_001.pdf]

SUPPORTING INFORMATION

Effect of iron doping in ordered nickel oxide thin film catalyst for the oxygen evolution reaction

Ane Etxebarria, Mauricio Lopez Luna, Andrea Martini, Uta Hejral, Martina Rüscher, Chao Zhan, Antonia Herzog, Afshan Jamshaid, David Kordus, Arno Bergmann, Helmut Kuhlenbeck, Beatriz Roldan Cuenya**

Department of Interface Science, Fritz-Haber Institute of the Max Planck Society, Faradayweg 4-6, 14195 Berlin, Germany

*etxebarria@fhi-berlin.mpg.de

*roldan@fhi-berlin.mpg.de

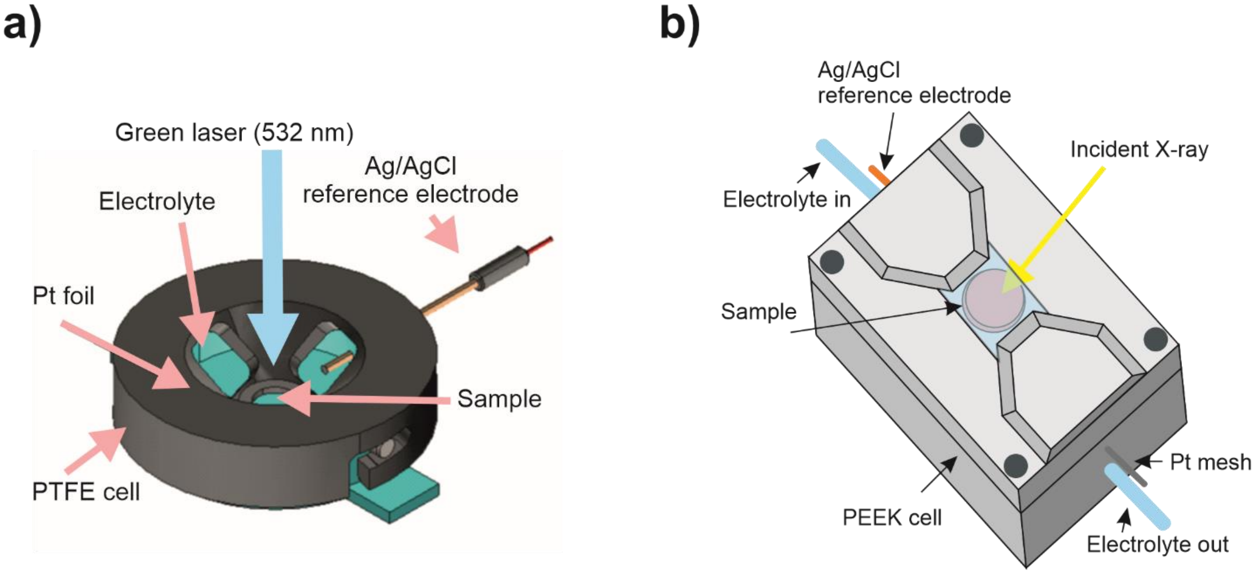

Figure S1. Simplified sketches of the *operando* (a) Raman and (b) grazing incidence X-ray absorption spectroscopy electrochemical cells used in this work.

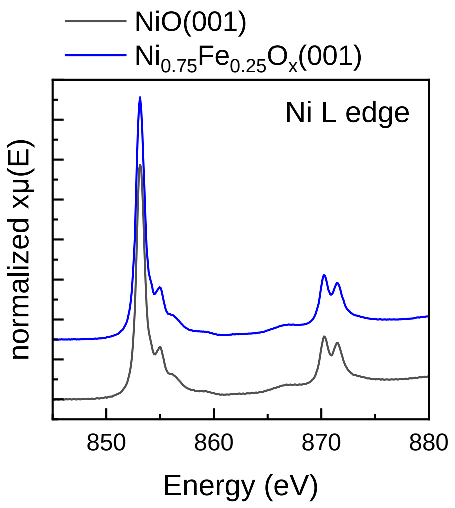

Figure S2. Ni L edge spectra of NiO(001) and $Ni_{0.75}Fe_{0.25}O_x$(001). The spectra correspond to that of NiO in rock salt structure.[1]

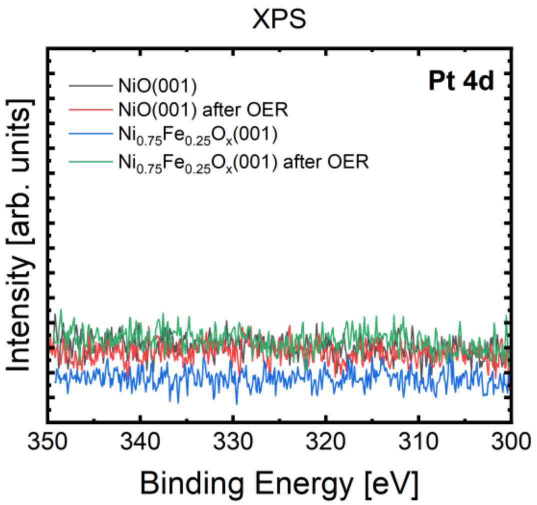

Figure S3. Pt 4d XPS spectra measured with Al K_α before and after a 2-hour OER treatment in Fe-purified 0.1 M KOH of NiO(001) and $Ni_{0.75}Fe_{0.25}O_x(001)$. The data reveal the absence of Pt on the sample surface.

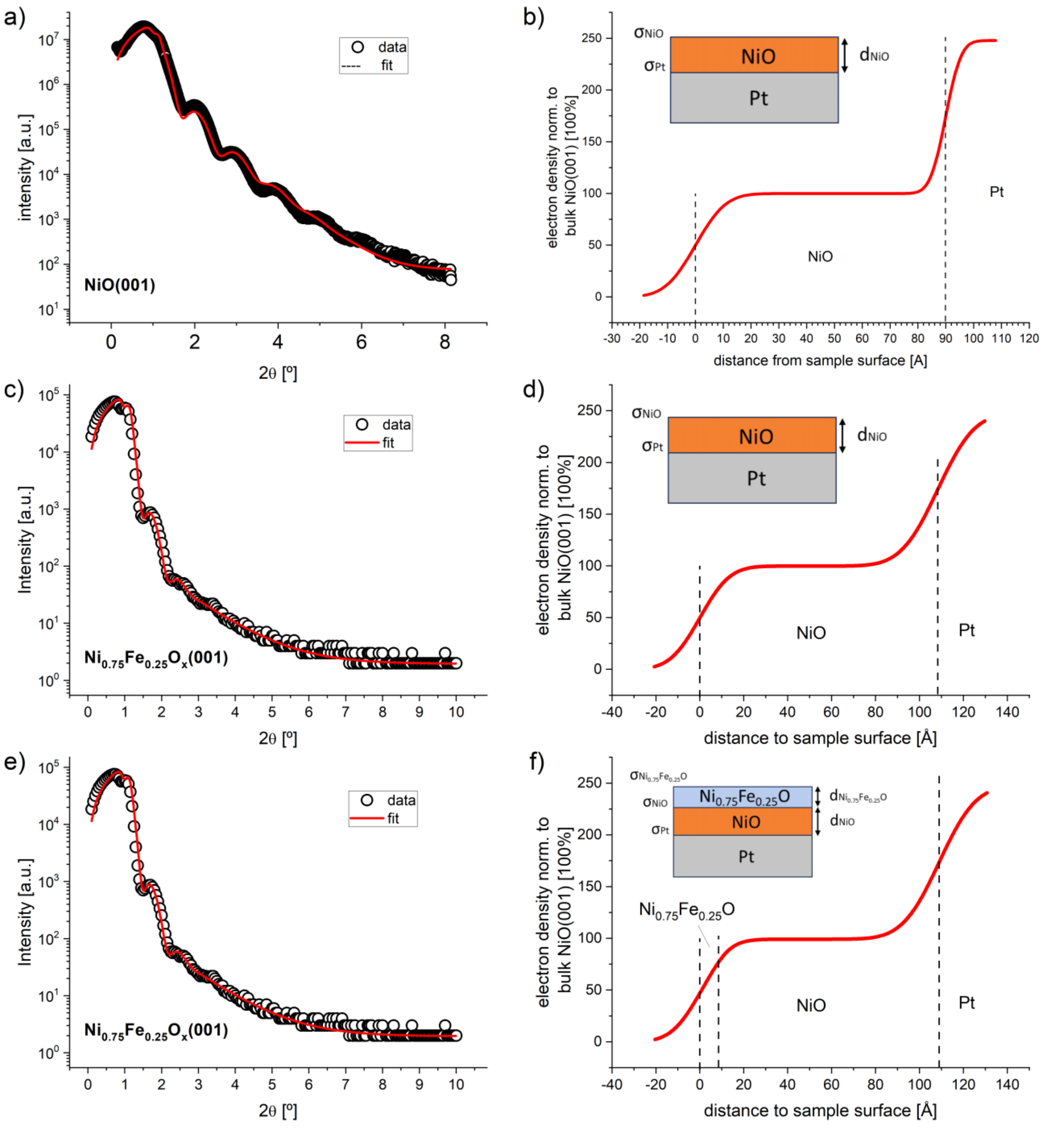

Figure S4. (a) XRR measurements performed on the as-prepared NiO(001)/Pt and (c,e) $Ni_{0.75}Fe_{0.25}O_x$(001)/Pt systems (open circles: experimental data, red solid line: fit to the data). (b) Electron density profiles as a function of the distance from the catalyst surface of NiO(001)/Pt, and (d,f) $Ni_{0.75}Fe_{0.25}O_x$/Pt systems. The inset in (b), (d), and (f) shows the underlying fit model used in (a), (c), and (e), respectively. The deduced thin film fit parameters are summarized in Tables S2-4.

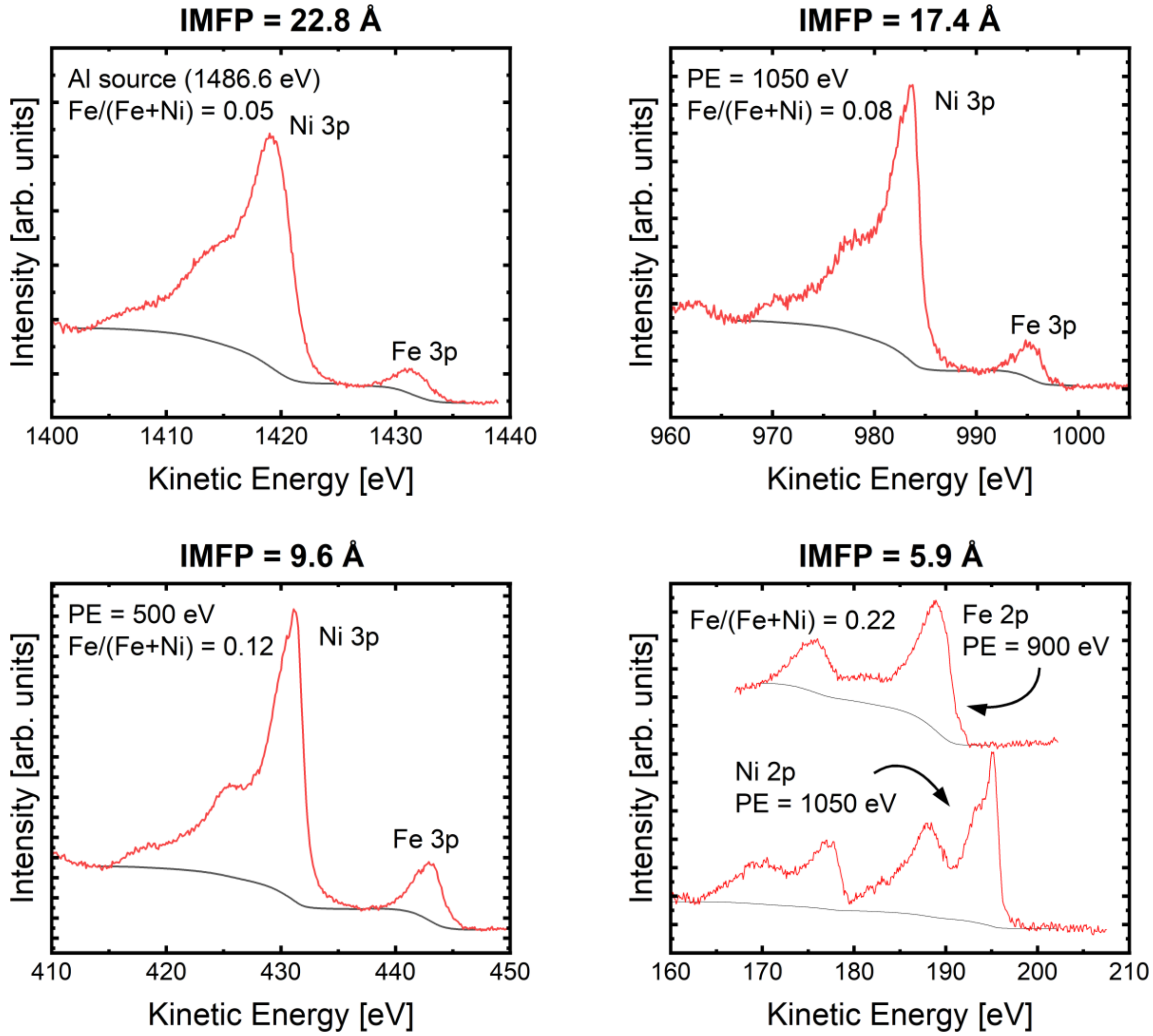

Figure S5. Ni and Fe XPS spectra measured with several photon energies (PE) of the as-prepared $Ni_{0.75}Fe_{0.25}O_x(001)$. The X-ray source and photon energies used, and the calculated iron concentration from the region of nickel and iron are indicated in each figure, as well as the Inelastic Mean Free Path (IMFP) values, which were obtained from the software QUASES-IMFP-TPP2M.

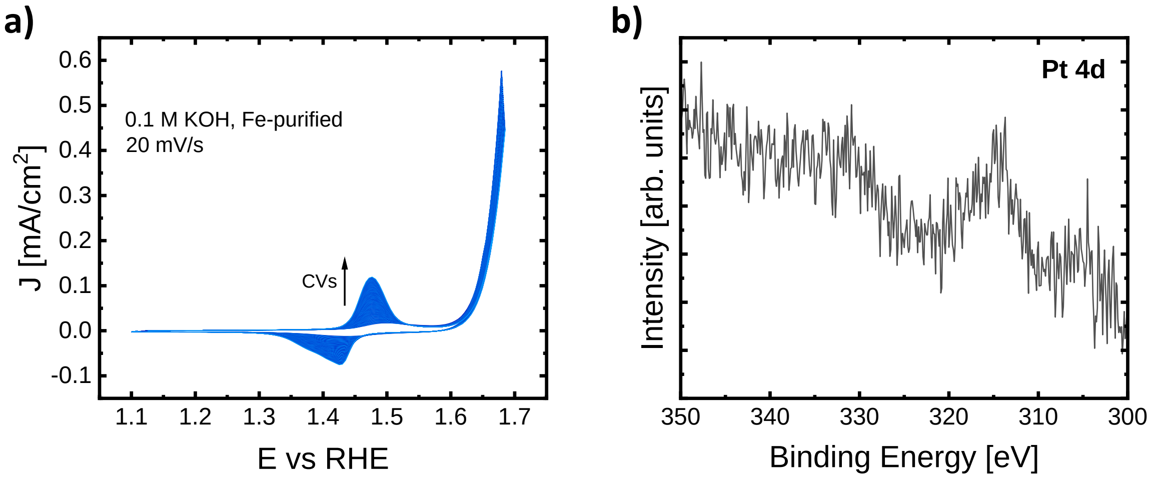

Figure S6. (a) 100 consecutive cyclic voltammetry cycles performed on an epitaxial NiO(001) in Fe-purified 0.1 M KOH. (b) Pt 4d XPS spectrum measured with Al Kα after 100 CVs in Fe-purified 0.1 M KOH of an epitaxial NiO(001). In (a), geometric current densities are shown.

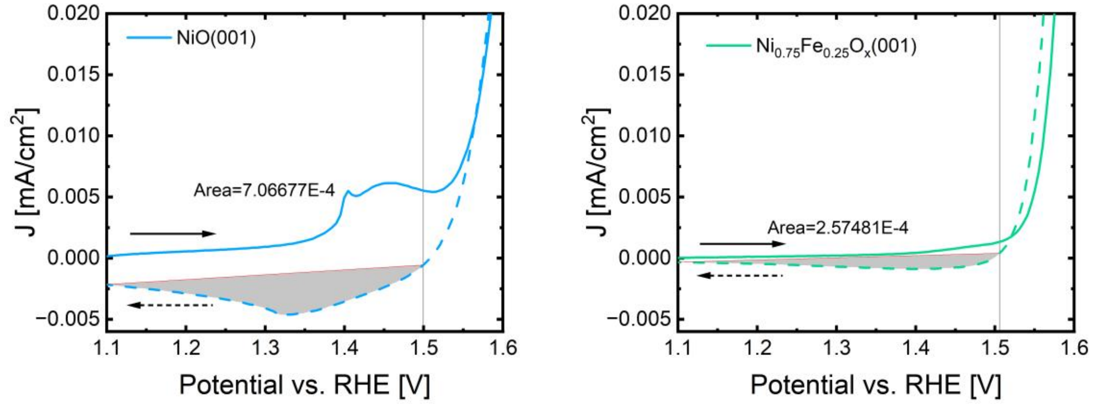

Figure S7. Anodic and cathodic linear sweep voltammetry after 2 hours of OER in Fe-purified 0.1 M KOH of NiO(001) and $Ni_{0.75}Fe_{0.25}O_x$(001). The area of the reduction of nickel is highlighted.

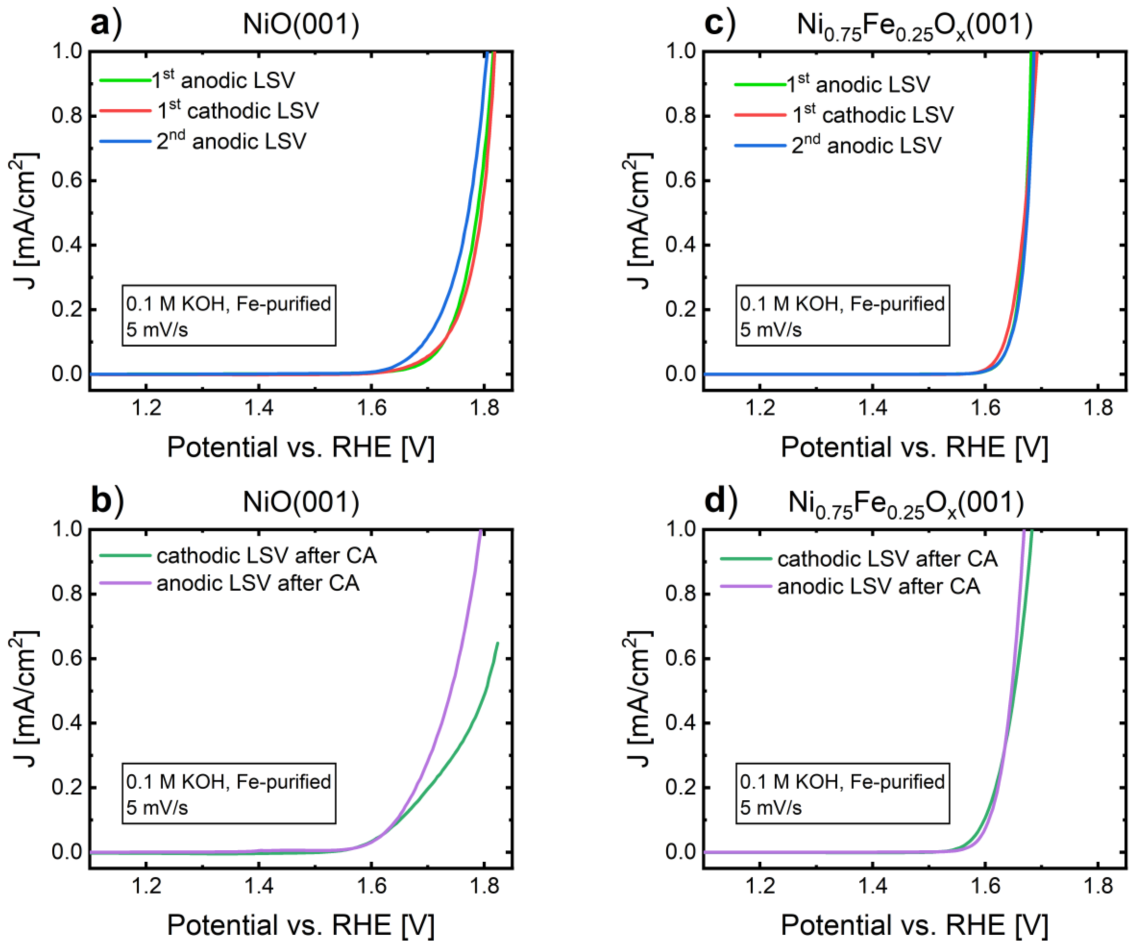

Figure S8. Linear sweep voltammograms (LSVs) of (a,b) NiO(001) and (c,d) $Ni_{0.75}Fe_{0.25}O_x$(001). The data in (a,c) were recorded at the beginning of the experiment up to 1 mA/cm^2, and the data in (b,d) after 2 h at OER conditions starting with a cathodic sweep.

OER on a disordered NiO thin film catalyst. A disordered NiO thin film was grown on a Ni(001) single crystal (MaTeck GmbH, Germany) by repetitive oxidation cycles in a 2×10^{-5} mbar O_2 atmosphere at 570 K. Comparison of the Ni2$p_{3/2}$ XPS spectrum of the Ni(001) single crystal before (Figure S9a) and after (Figure S9b) reveals the surface oxidation. Based on the XPS surface sensitivity of the measured data, the estimated thickness of the resulting disordered thin film is ca. 6 nm. Due to the disappearance of the underlying Ni(001) LEED pattern (Figures S9c) upon oxidation (Figure S9d), and, contrary to a previous study by Bäumer et al.,[2] the lack of detectable LEED intensity coming from crystalline NiO, we defined this film as a disordered one. The electrochemical performance was studied by an initial CV followed by an anodic linear sweep voltammetry (LSV), measured until reaching a potential that corresponds to a current density of 1 mA/cm^2. This potential was kept for 40 minutes. After that, a CV was recorded.

Similar to the ordered epitaxial NiO(001) thin film grown on Pt (thickness ca. 9 nm), maintaining the disordered oxide (thickness ca. 6 nm) at a potential that achieves a current density of 1 mA/cm^2 enhances its activity (Figure S10a) and increases the redox activity of Ni (Figure S10b). Comparing the OER activity of the epitaxial NiO(001) thin film with that of the disordered NiO thin film (Figure S11), we find a higher activity for the disordered NiO. One could expect this behavior, as different NiO surface orientations in the pre-catalyst electrode exhibit varying activities,[3] and defects could also play a role in increasing the activity. It should be noted that for the disordered thin film, geometric current densities have been used, unlike the case of the epitaxial NiO(001) thin film, where current densities were corrected for surface roughness, which implies that the activities of the disordered NiO could be lower if adjusted for roughness. In any case, for the epitaxial NiO(001) thin film, the roughness-scaled area is nearly the same as the geometric area (Table S1 on the manuscript), making them comparable.

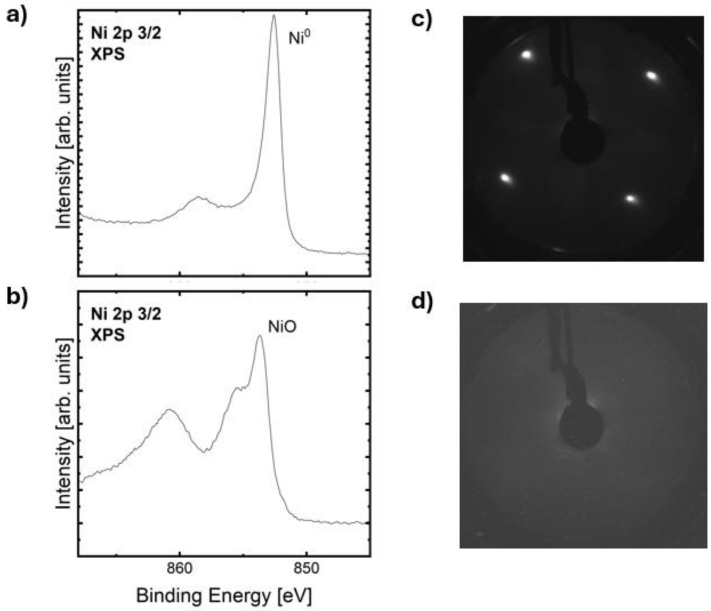

Figure S9. Ni 2$p_{3/2}$ XPS spectra measured with Al K_α (a,b) and LEED pattern measured at 65 eV (c,d) of a clean Ni(001) single crystal (a,c), and a disordered NiO thin film grown on the Ni(001) single crystal by oxidative treatments (b,d).

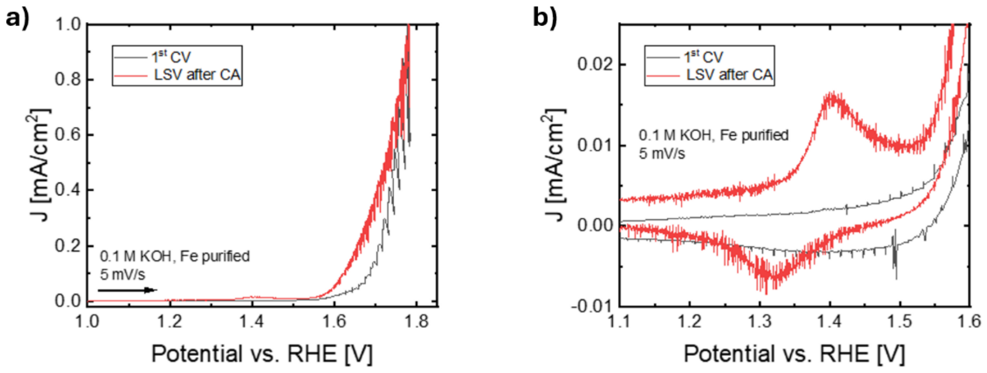

Figure S10. (a) Anodic LSV curves, and (b) nickel redox reaction area before and after a 40-minute OER treatment of a disordered NiO thin film grown on Ni(001). Geometric current densities are illustrated.

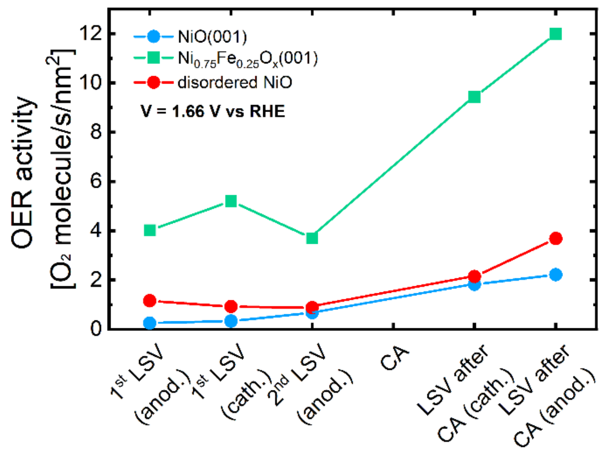

Figure S11. Oxygen evolution activities at 1.66V vs RHE at different states of the EC experiment for NiO(001) (thickness ca. 9 nm), $Ni_{0.75}Fe_{0.25}O_x$(001) (thickness ca. 11 nm), and a disordered NiO thin film (thickness ca. 6 nm), where "anod." means anodic, and "cath." means cathodic, related to the direction of the LSV scans. The CA was 2 hours long for NiO(001) and $Ni_{0.75}Fe_{0.25}O_x$(001), and 40 minutes long for the disordered NiO. For this last catalyst, the geometric area was used to estimate the OER activity, whereas for NiO(001) and $Ni_{0.75}Fe_{0.25}O_x$(001), the roughness-corrected area was used.

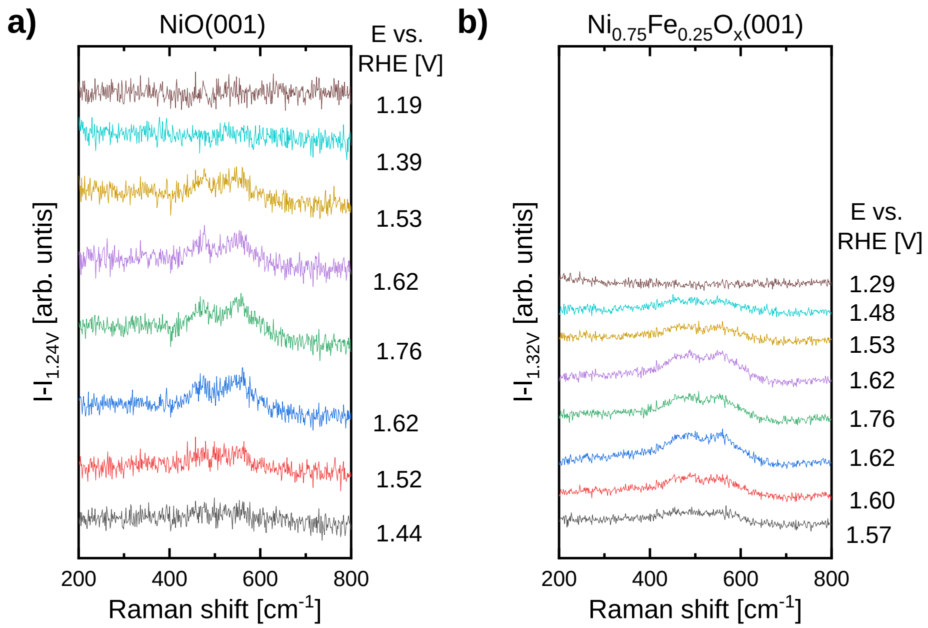

Figure S12. Operando Raman spectroscopy measurements of epitaxial (a) NiO(001) and (b) $Ni_{0.75}Fe_{0.25}O_x$(001) pre-catalysts. Spectra before the redox reaction have been subtracted from the spectra shown in the figure.

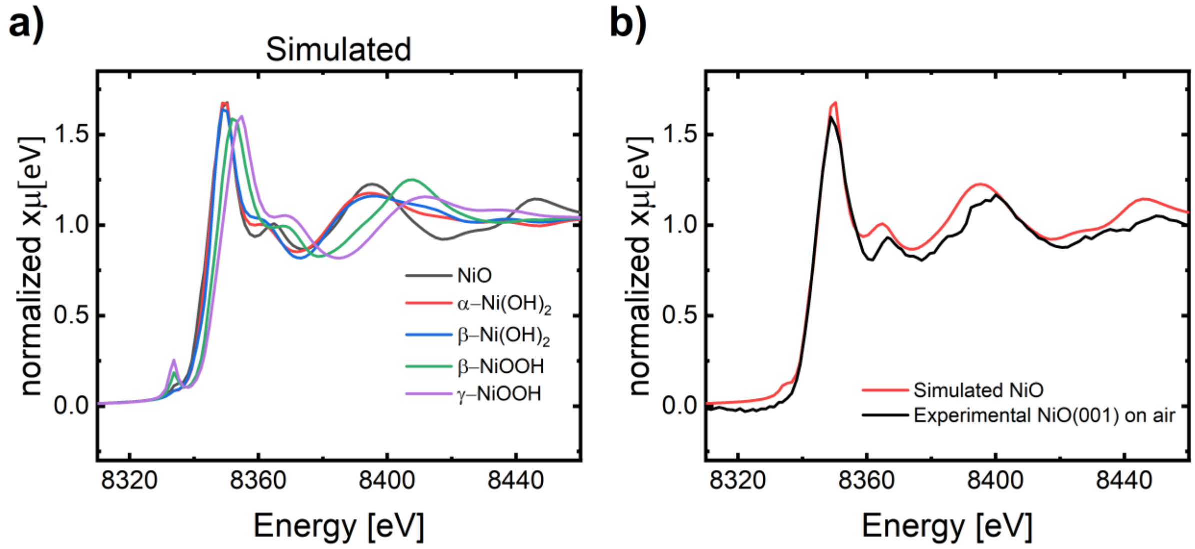

Figure S13. (a) Simulated XANES spectra of various compounds. (b) Comparison of simulated (using the FDMNES code) and experimental XANES spectra of NiO(001) thin film acquired in air.

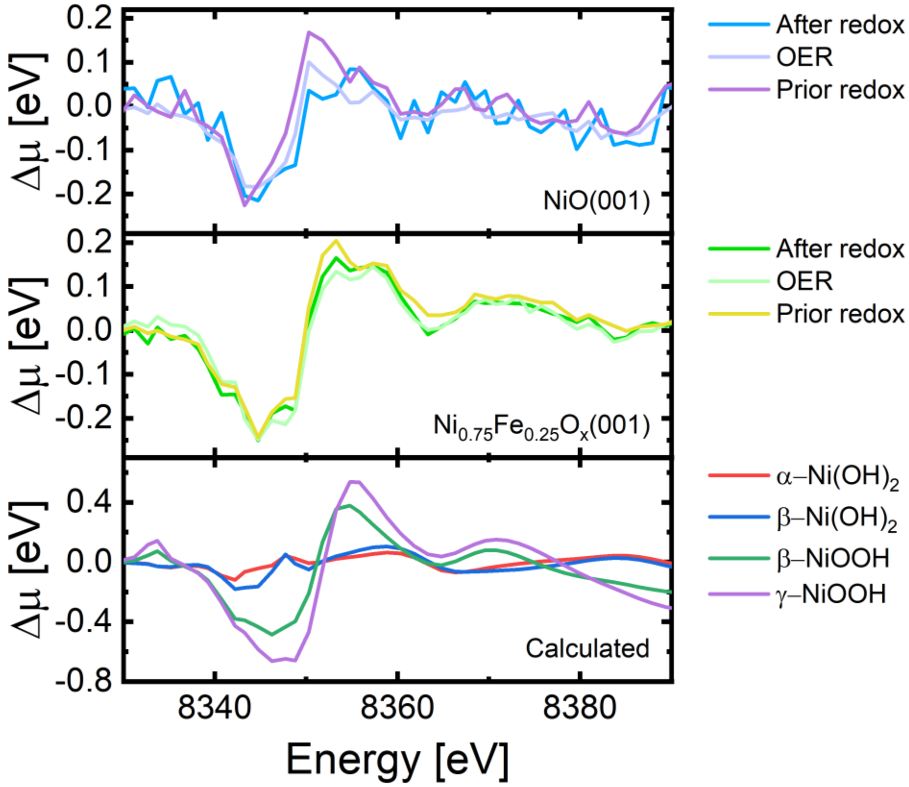

Figure S14. Δμ XANES spectra at several conditions of NiO(001) (top) and $Ni_{0.75}Fe_{0.25}O_x$(001) (middle). Theoretical Δμ XANES spectra (obtained from the profiles shown in Figure S13a of possible species that could be formed on NiO under reaction conditions (bottom).

Potentials measured during GIXAS experiments

- Above redox: 1.58 V vs. RHE for NiO(001), 1.57 V vs. RHE for $Ni_{0.75}Fe_{0.25}O_x(001)$.
- Under OER conditions: 1.68 V vs. RHE for NiO(001), 1.70 V vs. RHE for $Ni_{0.75}Fe_{0.25}O_x(001)$.
- After OER: 1.13 V vs RHE for NiO(001), 1.27 V vs RHE for $Ni_{0.75}Fe_{0.25}O_x(001)$.

Ni K-edge XANES simulations

Ni K-edge XANES simulations were realized using the finite difference approach (FDM), implemented in the FDMNES code.[4] The energy mesh used for spectra simulations featured an energy step of 0.02 eV near the Fermi level, while 2 eV and 30 eV above it. We performed the calculations using the real Hedin-Lundqvist[5] and von Barth[6] local exchange-correlation potential. In the spherical region around the atoms and the outer sphere, the electrostatic potential and the electron wave function were expanded in a series of spherical harmonics choosing the maximum value of the angular momentum l as $kr = \sqrt{l_{max}(l_{max}+1)}$, where k is the photoelectron wave vector and r is the radius of the sphere. The FDM computed cross sections were convoluted by means of an energy dependent function $\Gamma(\mathrm{E})$ defined as: $\Gamma(E) = \Gamma_{Hole} + \Gamma_{max}\left(\frac{1}{2} + \frac{1}{\pi}\arctan\left(\frac{\pi}{3}\frac{\Gamma_{max}}{E_l}\left(e - \frac{1}{e^2}\right)\right)\right)$, with $e = \frac{E-E_F}{E_c}$. In this expression Γ_{Hole} is the core-hole lifetime value, E_F is the Fermi energy, Γ_{max} is the maximum width associated to the final states while E_l and E_c correspond to the center and to the width of the arctangent function, respectively.

The following FDMNES parameters[7] were used throughout for the arctangent convolution: $\Gamma_{Hole} = 2.80\ eV$, $E_F = 8333\ eV$, $\Gamma_{max} = 17\ eV$, $E_c = 36\ eV$, $E_l = 40\ eV$.

The simulated spectra were then aligned by correcting each energy grid by their related EPSII parameter (corresponding to the energy required to bring one 1s core electron to the continuum for each structure). At the same time, we applied a common shift of 150 eV to all the simulated spectra. The latter was estimated aligning by the FDMNES simulated NiO spectrum to the Ni K-edge XANES of the NiO(001) acquired in air.

Table S1. The scaling factor of the surface areas of the thin films obtained from at least three 1x1 μm^2 AFM images, calculated using the Gwyddion software. The factor corresponds to the ratio between the surface area, which considers the roughness of the thin film, and the projected area, which refers to the AFM scan area. Error corresponds to the standard deviation calculated across the AFM images.

	**Scaling factor**	
**Thin Film**	**Before OER**	**After OER**
NiO(001)	1.0039 ± 0.0004	1.0035 ± 0.0006
$Ni_{0.75}Fe_{0.25}O_x$(001)	1.0073 ± 0.0008	1.0081 ± 0.0008

Table S2. XRR fit parameters obtained for the NiO/Pt catalyst, see the inset of Figure S4b for the underlying fit model. The fit parameter δ contributes to the real part of the refractive index via $n=1-\delta+i\beta$ and is a measure for the electron density, and thus the coverage, of the thin film.

**Fit parameter**	**Fitted value**	**Min. value of fitting range**	**Max. value of fitting range**
Surface root mean square roughness σ_{NiO} [Å]	8.62	$1.00 \cdot 10^{-2}$	$1.00 \cdot 10^{3}$
Layer thickness d_{NiO} [Å]	89.91	1.00	$1.00 \cdot 10^{3}$
2 δ_{NiO}	$4.21 \cdot 10^{-5}$	$1.00 \cdot 10^{-9}$	$4.21 \cdot 10^{-5}$
Interface root mean square roughness σ_{Pt} [Å]	4.39	$1.00 \cdot 10^{-2}$	$1 \cdot 10^{2}$

Table S3. XRR fit parameters obtained for the $Ni_{0.75}Fe_{0.25}O_x$/Pt catalyst, see the inset of Figure S4d for the underlying fit model. The fit parameter δ contributes to the real part of the refractive index via $n=1-\delta+i\beta$ and is a measure for the electron density, and thus the coverage, of the thin film.

**Fit parameter**	**Fitted value**	**Min. value of fitting range**	**Max. value of fitting range**
Surface root mean square roughness σ_{NiO} [Å]	10.74	$1.00 \cdot 10^{-2}$	$1.00 \cdot 10^{3}$
Layer thickness d_{NiO} [Å]	108.33	1.00	$1.00 \cdot 10^{3}$
2 δ_{NiO}	$4.20 \cdot 10^{-5}$	$1.00 \cdot 10^{-9}$	$4.21 \cdot 10^{-5}$
Interface root mean square roughness σ_{Pt} [Å]	13.20	$1.00 \cdot 10^{-2}$	$1 \cdot 10^{2}$

Table S4. XRR fit parameters obtained for the $Ni_{0.75}Fe_{0.25}O_x$/Pt catalyst, see the inset of Figure S4f for the underlying fit model. The fit parameter δ contributes to the real part of the refractive index via n=1-δ+iβ and is a measure for the electron density, and thus the coverage, of the thin film.

**Fit parameter**	**Fitted value**	**Min. value of fitting range**	**Max. value of fitting range**
Surface root mean square roughness $\sigma_{Ni0.75Fe0.25O}$ [Å]	10.44	$1.00\cdot10^{-2}$	$1.00\cdot10^{3}$
Layer thickness $d_{Ni0.75Fe0.25O}$ [Å]	8.55	1.00	$1.00\cdot10^{3}$
2 $\delta_{Ni0.75Fe0.25O}$	$3.95\cdot10^{-5}$	$1.00\cdot10^{-9}$	$4.04\cdot10^{-5}$
Interface root mean square roughness σ_{NiO} [Å]	4.41	$1.00\cdot10^{-2}$	$1.00\cdot10^{3}$
Layer thickness d_{NiO} [Å]	100.40	1.00	$1.00\cdot10^{3}$
2 δ_{NiO}	$4.17\cdot10^{-5}$	$1.00\cdot10^{-9}$	$4.21\cdot10^{-5}$
Interface root mean square roughness σ_{Pt} [Å]	13.20	$1.00\cdot10^{-2}$	$1\cdot10^{2}$

REFERENCES

(1) Chen, C. L.; Chern, G.; Pan, W. L.; Tseng, P. K.; Chang, C. L. Soft X-Ray Absorption Spectroscopy Studies of Single Crystalline Fe-Ni-O Alloy Thin Films. *J Electron Spectros Relat Phenomena* **2005**, *144–147*, 921–923. https://doi.org/10.1016/j.elspec.2005.01.104.

(2) Bäumer, M.; Cappus, D.; Kuhlenbeck, H.; Freund, H. J.; Wilhelmi, G.; Brodde, A.; Neddermeye, H. The Structure of Thin NiO(100) Films Grown on Ni(100) as Determined by Low-Energy-Electron Diffraction and Scanning Tunneling Microscopy. *Surf Sci* **1991**, *253* (1–3), 116–128. https://doi.org/10.1016/0039-6028(91)90585-G.

(3) Poulain, R.; Klein, A.; Proost, J. Electrocatalytic Properties of (100)-, (110)-, and (111)-Oriented NiO Thin Films toward the Oxygen Evolution Reaction. *Journal of Physical Chemistry C* **2018**, *122* (39), 22252–22263. https://doi.org/10.1021/acs.jpcc.8b05790.

(4) Guda, S. A.; Guda, A. A.; Soldatov, M. A.; Lomachenko, K. A.; Bugaev, A. L.; Lamberti, C.; Gawelda, W.; Bressler, C.; Smolentsev, G.; Soldatov, A. V.; Joly, Y. Optimized Finite Difference Method for the Full-Potential XANES Simulations: Application to Molecular Adsorption Geometries in MOFs and Metal-Ligand Intersystem Crossing Transients. *J Chem Theory Comput* **2015**, *11* (9), 4512–4521. https://doi.org/10.1021/acs.jctc.5b00327.

(5) Hedin, L.; Lundqvist, S. Effects of Electron-Electron and Electron-Phonon Interactions on the One-Electron States of Solids. *Solid State Physics* **1970**, *23*, 1–181.

(6) Von Barth, U.; Hedin, L. *A Local Exchange-Correlation Potential for the Spin Polarized Case: I*; 1972; Vol. 5.

(7) Joly, Y. FDMNES Manual. http://fdmnes.neel.cnrs.fr.
